# InceptionV4 and SEResNet101: precise predictors of intracranial hemorrhage and collateral circulation post—ischemic stroke intervention

**DOI:** 10.3389/fneur.2025.1617626

**Published:** 2025-09-17

**Authors:** Jing Zhang, Huawei Shen, Leping Zhou, Lihui Fu, Ting Song, Shuihua Zhang

**Affiliations:** ^1^Department of Radiology, Guangdong Provincial Key Laboratory of Major Obstetric Diseases; Guangdong Provincial Clinical Research Center for Obstetrics and Gynecology; The Third Affiliated Hospital of Guangzhou Medical University, Guangzhou, China; ^2^Department of Neurology, Guangdong Provincial Key Laboratory of Major Obstetric Diseases; Guangdong Provincial Clinical Research Center for Obstetrics and Gynecology; The Third Affiliated Hospital of Guangzhou Medical University, Guangzhou, China; ^3^Guangzhou Universal Medical Imaging Diagnostic Center, Guangzhou, China

**Keywords:** ischemic stroke, intracranial hemorrhage, collateral circulation, deep learning, computed tomography

## Abstract

**Background:**

Ischemic stroke (IS) is a major global health issue. The risk of intracranial hemorrhage (ICH) after interventional treatment and the status of collateral circulation significantly affect patient prognosis. Traditional diagnostic methods for predicting ICH and collateral circulation are limited. This study aimed to develop a more accurate prediction method using deep learning (DL) models.

**Methods:**

A meta-analysis was conducted on relevant literature. Five DL models (DenseNet169, InceptionResNetV2, InceptionV4, MobileNetV3Small, and SEResNet101) were trained and tested with preoperative CT images from 58 patients and the CQ500 dataset. An MCAO mouse model was established to identify biomarkers.

**Results:**

AI showed high accuracy in predicting ICH from CT images. InceptionV4 and SEResNet101 outperformed other models in diagnosing ICH and collateral circulation. Kdr, Lcn2, and Pxn were identified as key biomarkers for ICH and poor collateral circulation.

**Conclusion:**

The InceptionV4 or SEResNet101 algorithm, when combined with preoperative CT imaging, enables accurate and rapid prediction of intracranial hemorrhage and collateral circulation following interventional treatment in patients with ischemic stroke. This study presents an effective approach that integrates evidence-based medicine, radiomics, and deep machine learning technologies.

## Introduction

Ischemic Stroke (IS) is a global health issue affecting millions of people each year (1, 2). As a type of cerebrovascular accident, it not only causes severe physical harm but also imposes a significant burden on patients’ families and society (3, 4). This problem is becoming increasingly pronounced with the aging population. Interventional therapy is one of the primary treatments for IS; however, the risk of intracranial hemorrhage (ICH) following such procedures has been a major concern in the medical community. ICH not only exacerbates the patient’s condition but also significantly impacts treatment outcomes and prognosis (5, 6). Therefore, accurately assessing the risk of ICH before interventional therapy is a critical clinical challenge. Currently, imaging techniques, such as head Computed Tomography (CT) scans, are the primary methods used to evaluate ICH risk. However, traditional diagnostic approaches often rely on the physician’s experience and subjective judgment, which can limit diagnostic accuracy and efficiency (7, 8). Moreover, due to the complexity of stroke lesions, traditional methods have limitations in detecting subtle and early-stage abnormalities (9–11). Therefore, developing a more precise and efficient method for predicting ICH is crucial for improving treatment outcomes and prognosis in stroke patients.

Traditional medical imaging techniques, such as head CT scans, play a crucial role in the diagnosis of IS (12). These imaging modalities provide essential information about brain tissue structure and vascular conditions, serving as a vital basis for clinical diagnosis and treatment decisions (13). However, as technology advances, the limitations of traditional imaging techniques in terms of diagnostic accuracy and efficiency have become increasingly apparent. Against this backdrop, the application of machine learning, particularly Deep Learning (DL), to medical image analysis has emerged as a prominent research focus. DL techniques, by analyzing large volumes of medical imaging data, can automatically identify and learn complex patterns within the images, thereby enhancing diagnostic accuracy and efficiency. For instance, training DL models to recognize features of ICH following IS can help clinicians make more accurate and timely diagnoses (14).

Recent studies have shown promising results in using artificial intelligence (AI) and DL technologies to diagnose ICH after IS. Multiple studies indicate that DL has the potential to improve the Sensitivity (SEN) and Specificity (SPE) of ICH diagnosis (15–17). These studies typically leverage large datasets of medical images to train and test DL models, aiming to achieve or surpass the performance of traditional diagnostic methods. However, these studies face limitations in dataset size and model generalization. The relatively small scale of datasets used in many studies may affect the generalizability and clinical applicability of the models. Additionally, variations in the quality and format of medical imaging data produced by different institutions and equipment pose challenges to the models’ generalization and stability. Therefore, further research is needed to validate the application of DL in diverse clinical settings and to optimize the models to accommodate various data characteristics and requirements.

This study introduces a novel approach by employing five DL models—DenseNet169, InceptionResNetV2, InceptionV4, MobileNetV3Small, and SEResNet101—to predict ICH and collateral circulation in patients following interventional treatment for IS. Notably, the InceptionV4 and SEResNet101 algorithms demonstrated exceptional performance in image recognition tasks, consistent with existing literature (18–20). By applying these advanced algorithms to head CT scans, our research not only enhances predictive accuracy but also significantly accelerates processing times, which is critical for timely clinical decision-making in emergency settings. Furthermore, we have emphasized the generalizability of these models by training and testing them on diverse datasets from various sources and formats, resulting in robust stability and adaptability across different clinical environments. These advancements underscore the innovative value and potential applications of our study in the field of medical imaging analysis.

Initially, we conducted a meta-analysis to summarize the performance of AI in diagnosing ICH using CT imaging. Building upon this foundation, we integrated multiple DL algorithms with preclinical head CT scans to accurately and rapidly predict post-interventional ICH and collateral circulation in IS patients. Additionally, we aim to identify key biomarkers indicative of ICH and poor collateral circulation following reperfusion in middle cerebral artery occlusion (MCAO) mouse models. Through these methodologies, our objective is to provide clinicians with more effective tools to improve the diagnosis and treatment of IS patients.

## Materials and methods

### Meta-analysis

Literature search and selection: A systematic search was conducted in PubMed, Embase, and The Cochrane Library using the keywords “intracranial hemorrhage,” “computerized tomography (CT),” and “machine learning.” To ensure the precise selection of target literature, additional search terms such as NOT (animal experiment OR meeting OR case report OR review) were applied. The literature search was conducted up until November 2023. Following the search, articles were rigorously screened according to predetermined inclusion and exclusion criteria.

Data extraction: The screening of literature was managed using the reference management software EndNote X9 (Clarivate Analytics, United States). After automatically and manually removing duplicates, two independent researchers screened the articles by title, abstract, and full text based on pre-established eligibility criteria. Data extraction focused on key outcome measures, including True Positive (TP), False Positive (FP), False Negative (FN), and True Negative (TN) values. Any discrepancies between the researchers were resolved through discussion with a third researcher. In cases where the studied populations overlapped, the study with the larger participant count was selected.

Quality assessment: The quality of the included studies was independently assessed by two researchers using the Quality Assessment of Diagnostic Accuracy Studies 2 (QUADAS-2) tool. In cases of disagreement, a third researcher provided an evaluation. QUADAS-2 comprises four domains: patient selection, index test, reference standard, and flow and timing. Each domain’s risk of bias was categorized as “low,” “high,” or “unclear” based on responses to relevant signaling questions. The R package “robvis” was utilized to generate a summary, and traffic light plots were used to visualize the quality assessment results.

Quantitative meta-analysis: Meta-analysis was performed using Stata 17 software, synthesizing the diagnostic results from all included studies. Forest plots were generated to display SEN, SPE, diagnostic score, and diagnostic odds ratio (DOR). Heterogeneity was assessed using the *Q* test and *I*^2^ statistic, considering *p* values and *I*^2^ values to determine the presence and magnitude of heterogeneity. Scatter plots were created for each included study, and the Summary Receiver Operating Characteristic (SROC) curve was fitted to assess the diagnostic value, evaluated by the area under the curve (AUC). Fagan’s nomogram was used to calculate post-test probabilities based on prior probabilities, reflecting the diagnostic value. Deek’s funnel plot was constructed to investigate potential publication bias, and scatter plots were used to evaluate the diagnostic performance of the included studies.

### Collection and grouping of clinical data

We collected clinical data from 58 patients diagnosed with IS who underwent interventional procedures at our hospital between January 2022 and March 2023. All patients underwent preoperative head CT scans. Detailed clinical information was gathered, including histories of diabetes, hypertension, hyperlipidemia, coronary artery disease, atrial fibrillation, and previous strokes. Additionally, preoperative and postoperative scores were recorded for the Thrombolysis in Cerebral Infarction (TICI), ICH, National Institutes of Health Stroke Scale (NIHSS), modified Rankin Scale (mRS), CT-ASPECTS, and Collateral scores. Three neuroradiologists jointly determined the diagnoses of ICH and collateral circulation. Patients were grouped based on their ICH and collateral circulation status. The ICH group was divided into two categories: Bleeding and Non-Bleeding. Based on the Collateral score, patients were classified into three groups: Good (3–4 points), Partial (2 points), and Poor (0–1 points).

The TICI criteria are defined as follows: 0 indicates no perfusion or antegrade flow beyond the occlusion site; 1 indicates that contrast medium passes through the occluded area on digital subtraction angiography (DSA), but the distal vascular bed is not visualized; 2 indicates partial reperfusion with visualization of the distal vascular bed, albeit slower than the healthy side; 3 indicates complete reperfusion with normal filling.

The ICH assessment criteria are as follows: Hemorrhagic infarction (HI) is categorized into HI1, which involves petechial hemorrhage, and HI2, which involves confluent hemorrhage without mass effect. Parenchymal hemorrhage (PH) is categorized into PH1, where the hematoma occupies less than 30% of the area with mass effect, and PH2, where the hematoma occupies more than 30% with mass effect. The scoring is as follows: HI1 = 1 point, HI2 = 2 points, PH1 = 3 points, and PH2 = 4 points.

The NIHSS score is used to evaluate the severity of cerebral infarction. A lower score indicates a milder condition, while a higher score suggests greater disability and functional decline caused by the infarction. Typically, a score of 3 or less indicates a mild stroke, 3–10 indicates a moderate stroke, and a score above 10 indicates a severe stroke.

The CT-ASPECTS score is specifically used to assess the effectiveness of intravenous thrombolysis in patients based on non-contrast CT scans. The scoring method involves selecting two CT slices and assigning values to different vascular territories, with each territory scoring 1 point. If an area shows an edematous low-density lesion, 1 point is deducted.

The mRS scoring criteria are as follows: 0 indicates no symptoms; 1 indicates symptoms without significant functional impairment, allowing the completion of all usual activities; 2 indicates slight disability, where the patient cannot perform all pre-stroke activities but can take care of their own affairs without assistance; 3 indicates moderate disability, requiring some help but the patient can walk independently; 4 indicates moderately severe disability, where the patient cannot walk independently and requires help with daily activities; 5 indicates severe disability, bedridden, incontinent, and entirely dependent on others for care.

The collateral score is defined as follows: 0 = absent collaterals in more than 50% of the M2 territory; 1 = diminished collaterals in more than 50% of the M2 territory; 2 = diminished collaterals in less than 50% of the M2 territory; 3 = collaterals equal to the contralateral side; 4 = increased collaterals.

### Preprocessing of experimental data

Before extracting features and classifying the CT images, it is essential to preprocess the data. The preprocessing of CT images involves the following steps: (1) motion correction and format adjustment; (2) non-uniform intensity normalization using the N3 algorithm; (3) calculation of the Talairach transformation; (4) intensity normalization; (5) skull stripping and affine registration using FreeSurfer 3.4.0 software; (6) spatial normalization of the data to the Montreal Neurological Institute (MNI) space using Statistical Parametric Mapping (SPM); and (7) smoothing with an 8-mm Gaussian filter in MATLAB 2020a.

### Construction of deep machine learning models and image data analysis

We utilized the CUDA 10 parallel computing platform along with the CUDNN 7.3 deep neural network GPU acceleration library to establish the experimental environment. The functional modules were implemented using Python, and PyTorch 1.x was selected as the DL framework. The GPU used in the setup was an NVIDIA RTX 2080 Ti with 11GB of memory. The preprocessed data was imported, and Magnetic Resonance Imaging (MRI) data was normalized. For ICH detection, the CQ500 dataset (http://headctstudy.qure.ai/) was chosen as the test dataset. The CQ500 dataset is a publicly available collection of head CT scans specifically designed for ICH detection. It comprises 491 head CT scans, encompassing over 193,000 individual CT slices, and is primarily used to train and evaluate DL models in detecting intracranial pathologies such as hemorrhages, fractures, and infarctions. Each CT scan in the CQ500 dataset is annotated by multiple radiologists, providing detailed information on the location and type of lesions, ensuring the dataset’s high quality and accuracy.

For collateral circulation assessment, only non-contrast-enhanced baseline CT data (pre-intervention) was used, excluding CT perfusion images. Specifically, 20% of the CT images from 58 IS patients were randomly selected as an independent test set, while the remaining data was used for model training. The data was subjected to training and classification tests to develop a predictive model. The model’s accuracy, SEN, and SPE were calculated on the test set.

The general process of deep machine learning model construction is illustrated in Supplementary Figure S1.

In this study, we employed five DL models: DenseNet169, InceptionResNetV2, InceptionV4, MobileNetV3Small, and SEResNet101.

The Dense Convolutional Network (DenseNet) 169 addresses the vanishing gradient problem by utilizing a simple connectivity pattern that ensures maximal information flow between layers during both forward and backward computations. In this architecture, each layer receives inputs from all preceding layers, passing its own feature maps to all subsequent layers. DenseNet consists of three dense blocks, with transition layers between adjacent blocks that adjust the size of feature maps through convolution and pooling operations (21) (Supplementary Figure S2). The model used in this study comprises 169 layers.

The InceptionResNetV2 is a convolutional neural network (CNN) that combines the structures of Inception (also known as Network-in-Network) and Residual Network (ResNet). The key feature of InceptionResNetV2 lies in its integration of the Inception and ResNet architectures, which is designed to enhance both the performance and stability of the model. The Inception module excels at extracting features across multiple scales, while the residual connections facilitate the learning of complex mappings, thereby mitigating the issue of vanishing gradients commonly encountered in deep network training (Supplementary Figure S3).

InceptionV4 is an advanced modification of the earlier versions of GoogLeNet. This revised structure significantly improves the overall algorithm’s effectiveness. The introduction of parallel structures in the Stem section and the fusion of Residual blocks with the Inception module has led to further performance breakthroughs (22) (Supplementary Figure S4).

MobileNet, a lightweight DL network architecture, was introduced by Google’s research team in 2017. The key innovation of MobileNet lies in its use of depthwise separable convolutions, which replace traditional convolution operations. Depthwise separable convolutions consist of two steps: depthwise convolution, which applies convolution independently to each channel, and pointwise convolution, which uses 1 × 1 convolutions to alter the number of channels. This approach significantly reduces the model’s parameters and computational complexity, making MobileNet suitable for deployment on resource-constrained devices. With continued research, MobileNet has evolved into multiple versions, such as MobileNetV3, which incorporates linear activation functions and residual connections. The overall structure of the large and small versions is consistent, with differences primarily in the number of bneck modules and internal parameters, particularly the channel count. This study employs MobileNetV3Small (Supplementary Figure S5).

Squeeze-and-Excitation Networks (SENet) focus on enhancing model performance by dynamically recalibrating channel-wise feature responses based on learned weights from the network. By assigning higher weights to more informative feature maps and lower weights to less effective ones, SENet aims to improve the overall results. Although integrating SE blocks into existing classification networks increases the model’s parameters and computational load, the performance gains often justify this trade-off. By embedding SE modules into the fundamental building blocks of original network structures, various SENet variants, such as SEResNet101 (Supplementary Figure S6), can be created. This study utilizes SEResNet101.

### Construction of animal models and grouping

SPF-grade adult male C57BL/6 mice (22–27 g; 8–10 weeks old) were purchased from our institution’s Laboratory Animal Center. The mice were housed in an SPF-grade facility with controlled humidity (45–50%) and temperature (25–27 °C) for 1 week under a 12-h light/dark cycle to acclimate to the experimental conditions. All animal procedures were approved by the Institutional Animal Care and Use Committee.

Focal cerebral ischemia was induced using a modified MCAO model. Briefly, the mice were anesthetized with a mixture of 3% isoflurane (792632, Sigma Aldrich, United States) and oxygen/nitrous oxide (30%:67%). A midline incision was made in the neck to expose the Common Carotid Artery (CCA), Internal Carotid Artery (ICA), and External Carotid Artery (ECA). A silicone-coated monofilament (0.2 mm in diameter) was inserted through the ECA into the ICA and advanced to the origin of the Middle Cerebral Artery (MCA) until Cerebral Blood Flow (CBF) was reduced to approximately 20% of the baseline. CBF was measured before MCAO, during MCAO, and after reperfusion using a laser speckle contrast imaging system (SIM BFI HR, Wuhan Simopto Photonics Technology Co., Ltd.). After 60 min of MCAO, the filament was removed to restore blood flow, thereby establishing the reperfusion (MCAO/R) model (23).

On the first day after reperfusion, CT Angiography (CTA) was performed on the mice to assess the status of ICH and collateral circulation. The CTA was conducted using a micro-CT scanner from GE Healthcare, with a contrast agent (300 mg/mL Iohexol, D2158, Sigma Aldrich, United States) administered via tail vein injection at a dose of 0.1 mL/10 g. The CT scan parameters were set to a voltage of 50 kV, a current of 0.5 mA, and a slice thickness of 1 mm. Intracranial arteries and collateral circulation were evaluated using multiphasic CT Angiography (mCTA). Based on the presence of ICH, the MCAO mice were divided into two groups: Non-Bleeding and Bleeding. Additionally, the MCAO mice were categorized into Good and Poor groups according to the collateral circulation status. Specifically, the Good group exhibited well-developed collateral vessels and sufficient blood flow restoration, while the Poor group showed fewer or less prominent collateral vessels, leading to poor blood flow recovery and inadequate compensation of the ischemic area.

### Transcriptomic high-throughput sequencing

We collected brain tissue samples from the ischemic penumbra region of mice in the Non-Bleeding group (*n* = 3) and Bleeding group (*n* = 3), as well as in the Good group (*n* = 3) and Poor group (*n* = 3), for transcriptomic high-throughput sequencing. The detailed procedure is as follows: Total RNA was extracted from each sample using Trizol reagent (T9424, Sigma-Aldrich, United States) according to the manufacturer’s instructions. RNA concentration, purity, and integrity were measured using the Qubit^®^ RNA Assay Kit in the Qubit^®^ 2.0 Fluorometer (Q32852, Life Technologies, United States), a nanophotometer spectrophotometer (IMPLEN, Germany), and the RNA Nano 6000 Assay Kit in the Bioanalyzer 2100 system (5067-1512, Agilent, United States), respectively, with A260/280 ratios between 1.8 and 2.0. A total of 3 μg of RNA per sample was used as input material for RNA sample preparation. The complementary DNA (cDNA) libraries were generated using the NEBNext^®^ Ultra^™^ RNA Library Prep Kit for Illumina (E7760S, GENE COMPANY LIMITED, China) according to the manufacturer’s protocol and were evaluated for quality using the Agilent Bioanalyzer 2100 system. Clustering of the index-coded samples was performed on a cBot Cluster Generation System using the TruSeq PE Cluster Kit v3 cBot HS (Illumina) following the manufacturer’s recommendations. After cluster generation, the libraries were sequenced on the Illumina HiSeq 550 platform, producing 125 bp/150 bp paired-end reads.

### Quality control and analysis of sequencing data

The quality of the paired-end reads from the raw sequencing data was assessed using FastQC (v0.11.8). Preprocessing of the raw data was performed using Cutadapt (v1.18) to remove Illumina sequencing adapters and poly(A) tail sequences. Reads with an N content exceeding 5% were eliminated using a custom Perl script. The FASTX Toolkit (v0.0.13) was used to extract reads with at least 70% of their bases having a quality score above 20. Paired-end sequences were then repaired using BBMap (v39.01). Finally, the filtered high-quality reads were aligned to the human reference genome using HISAT2 (v0.7.12).

Based on the transcriptome high-throughput sequencing data, differential expression analysis was conducted using the R package limma. Genes with a |log_2_FC| > 1 and an adjusted *p*-value (adj*p*) < 0.05 were identified as differentially expressed genes (DEGs) between the Non-Bleeding and Bleeding groups, as well as between the Good and Poor groups.

To further elucidate the specific functions of the DEGs, we performed gene ontology (GO) and Kyoto Encyclopedia of Genes and Genomes (KEGG) enrichment analyses using the R package clusterProfiler, with a significance threshold of *p* < 0.05. The GO categories included biological processes (BP), cellular components (CC), and molecular functions (MF). Results were visualized using lollipop plots.

### Immunofluorescence staining

Brain tissue samples from mice were collected at 1, 3, and 7 days after reperfusion. These samples were processed into sections following standard procedures. The sections were fixed in cold 4% paraformaldehyde (60536ES60, Yeasen Biotechnology, China) for 15 min, then washed and permeabilized using 0.25% Triton-X100/PBS solution (P0096, Beyotime, China). Subsequently, the sections were incubated overnight at 4 °C with primary antibodies: CD31 (ab222783, 1:100, Abcam, UK) and ZO-1 (ab307799, 1:500, Abcam, UK). After three washes with PBS, the sections were incubated for 1 h with Alexa Fluor 488-conjugated secondary antibodies (ab150129/ab150077, 1:200, Abcam, UK). The sections were then washed three more times with PBS and stained with 4′,6-diamidino-2-phenylindole (DAPI) (10 μg/mL, D3571, Thermo Fisher, United States) for 10 min at room temperature. The sections were stored at 4 °C and subsequently observed using a fluorescence microscope (IMT-2, Olympus).

### Cell culture

Primary mouse brain microvascular endothelial cells (mBMECs) were isolated from 10-day-old C57BL/6 mice. Brain tissue was first dissected, and the gray matter was cut into small pieces. A 25% BSA solution was added to the tissue suspension, followed by homogenization and centrifugation at 600 × g for 10 min. The microvascular pellet at the bottom was collected and digested with 0.1% type II collagenase at 37 °C for 35 min. The resulting microvascular fragments and endothelial cells were cultured in a DMEM/F12 medium containing 20% fetal bovine serum (FBS) (F8687, Sigma-Aldrich, United States). Once mBMECs reached 90% confluence, the medium was replaced with endothelial cell medium (1001, Sciencell, USA) supplemented with 5% FBS, 1% endothelial cell growth supplement, and 1% penicillin/streptomycin. All cells were maintained at 37 °C in a humidified atmosphere with 5% CO_2_, and the culture medium was refreshed every 2–3 days.

### The oxygen–glucose deprivation and oxygen–glucose deprivation/reoxygenation models

The *in vitro* IS model was established by combining oxygen–glucose deprivation (OGD) and oxygen–glucose deprivation/reoxygenation (OGD/R) experiments. Briefly, after culturing the mBMECs to 90–95% confluence for 7–9 days, the neurons were washed three times with PBS and then placed in an anaerobic chamber containing glucose-free DMEM (11966-025, ThermoFisher, United States) with 5% CO_2_ and 95% N_2_. The cells were incubated at 37 °C for 24 h. Following this, the medium was replaced with a normal culture medium, and the cells were maintained in a 37 °C incubator with 5% CO_2_ for 6, 24, or 72 h to allow for reoxygenation. Cells in the control group were cultured under normal conditions with a complete medium (24).

### Real-time quantitative polymerase chain reaction

Total RNA was extracted from tissues and cells using TRIzol reagent (15596026, ThermoFisher, United States), and the concentration and purity of the extracted RNA were measured using a Nanodrop 2000 spectrophotometer (ThermoFisher, United States). cDNA was synthesized from mRNA following the instructions of the PrimeScript RT reagent Kit (Takara Code: RR047A, Takara, Japan), with gene-specific primers synthesized by TaKaRa (Supplementary Table S1). Real-time quantitative polymerase chain reaction (RT-qPCR) was performed on a 7,500 Fast Real-Time PCR System (4351106, ThermoFisher, United States), using Gapdh as the internal reference gene. The relative expression levels of the target genes were calculated using the 2^-ΔΔCt^ method, where ΔΔCt = ΔCt (experimental group) - ΔCt (control group) and ΔCt = Ct (target gene) - Ct (reference gene). The relative transcription level of the target gene mRNA was determined as 2^−ΔΔCt^. Each experiment was conducted in triplicate.

### Western blot

Total protein was extracted from cells or tissues using high-efficiency RIPA lysis buffer according to the manufacturer’s instructions. After lysis at 4 °C for 15 min, the samples were centrifuged at 12,000 × g for 15 min to collect the supernatant. Protein concentration in each sample was determined using a BCA protein assay kit (20201ES76, Yeasen Biotechnology, China). Equal amounts of protein were subjected to polyacrylamide gel electrophoresis and subsequently transferred onto PVDF membranes using the wet transfer method. The membranes were blocked with 5% BSA at room temperature for 1 h, followed by overnight incubation at 4 °C with primary antibodies (details in Supplementary Table S2). After washing the membranes three times with TBST for 5 min each, they were incubated with HRP-conjugated goat anti-rabbit IgG (ab205718, 1:20000, Abcam, UK) at room temperature for 1 h. The membranes were then washed again with TBST (3 × 5 min) and developed with an appropriate chemiluminescent substrate. Protein quantification was performed using ImageJ software (v1.48, National Institutes of Health) by calculating the ratio of the grayscale value of each target protein to that of the internal control, Gapdh. Each experiment was repeated three times.

### Statistical analysis

All statistical analyses in this study were conducted using Statistical Package for the Social Sciences (SPSS) software (version 21.0, IBM, United States). The data are presented as Mean ± SD. Comparisons between two normally distributed groups were performed using the unpaired Student’s *t*-test, while comparisons among multiple groups were conducted using one-way Analysis of Variance (ANOVA) followed by Tukey’s *post-hoc* test.

## Results

### AI has demonstrated high accuracy in the intelligent prediction of ICH on CT images

The application of AI in medical imaging analysis has advanced rapidly, particularly in the diagnosis of stroke. ICH and collateral circulation are critical factors for the prognosis of IS patients, yet traditional methods often struggle to accurately assess these conditions (17, 25). To evaluate the potential of AI, this study first conducted a meta-analysis to summarize the performance of AI in diagnosing ICH on CT images.

The literature search and selection process are illustrated in Supplementary Figure S7A. The process involved searching PubMed, Embase, and The Cochrane Library databases, yielding a total of 68 articles after removing duplicates. After excluding studies that were unrelated to ICH or CT imaging diagnosis, did not demonstrate the diagnostic effectiveness of DL models, or had incomplete data, 18 studies (encompassing 22 datasets) were selected for this meta-analysis.

All 18 studies utilized CNN as the DL method. The study designs included both prospective and retrospective approaches, with most studies employing a sequential or randomized design. The gold standard was the diagnosis by one or more radiologists or neuroradiologists (Supplementary Table S3). All included articles were full-text studies, and their quality was assessed using the QUADAS-2 tool, which indicated a low risk of bias in the selected domains for all studies (Supplementary Figures S7B,C).

**Figure 1:**
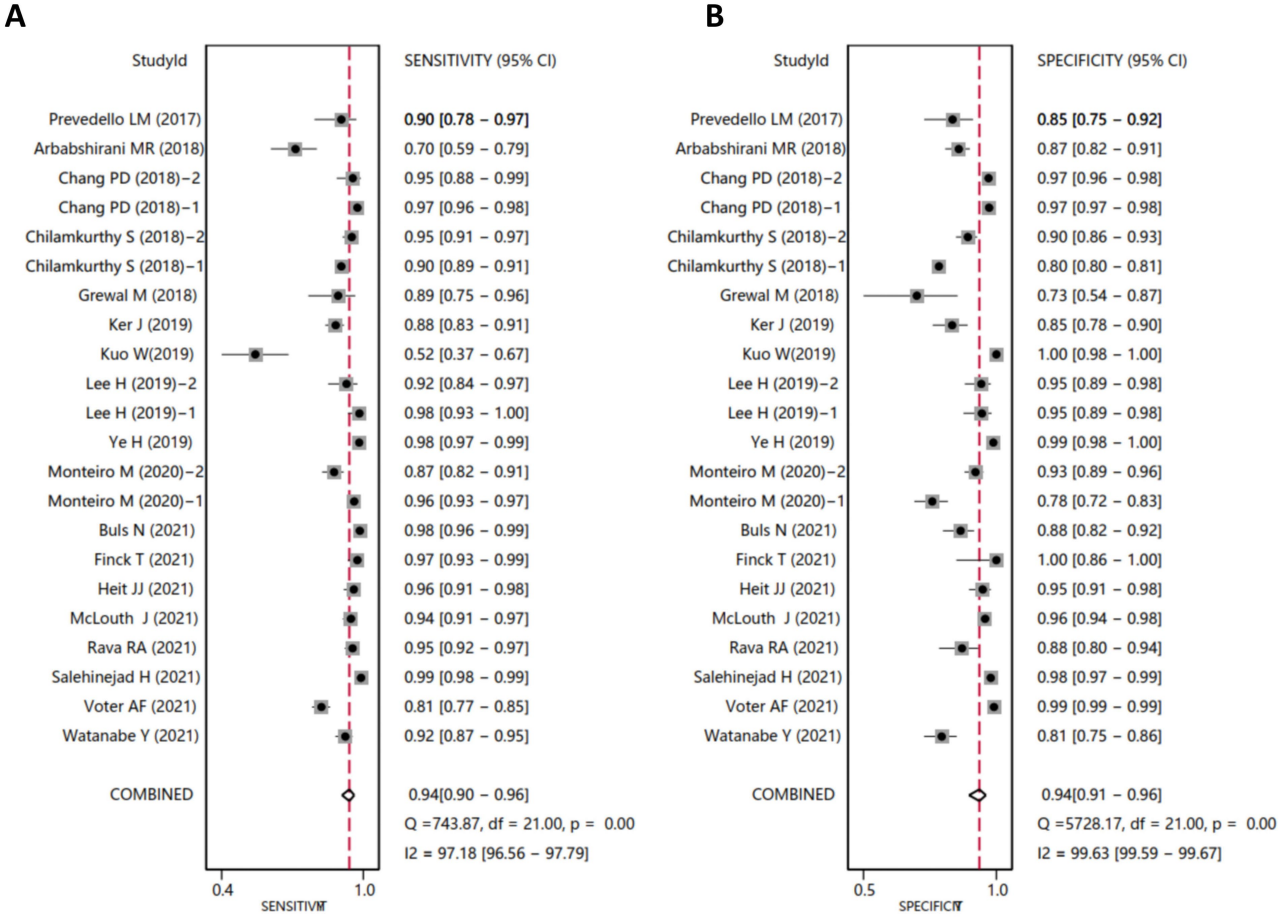
Forest plots of SEN and SPE for AI-assisted CT imaging in diagnosing ICH. (**A, B**) The *Q*-test yielded a *p*-value < 0.05, indicating statistically significant heterogeneity among the included studies. An *I*^2^ value greater than 50% suggests notable heterogeneity. The closer the SEN and SPE values are to 1, the higher the diagnostic accuracy.

**Figure 2:**
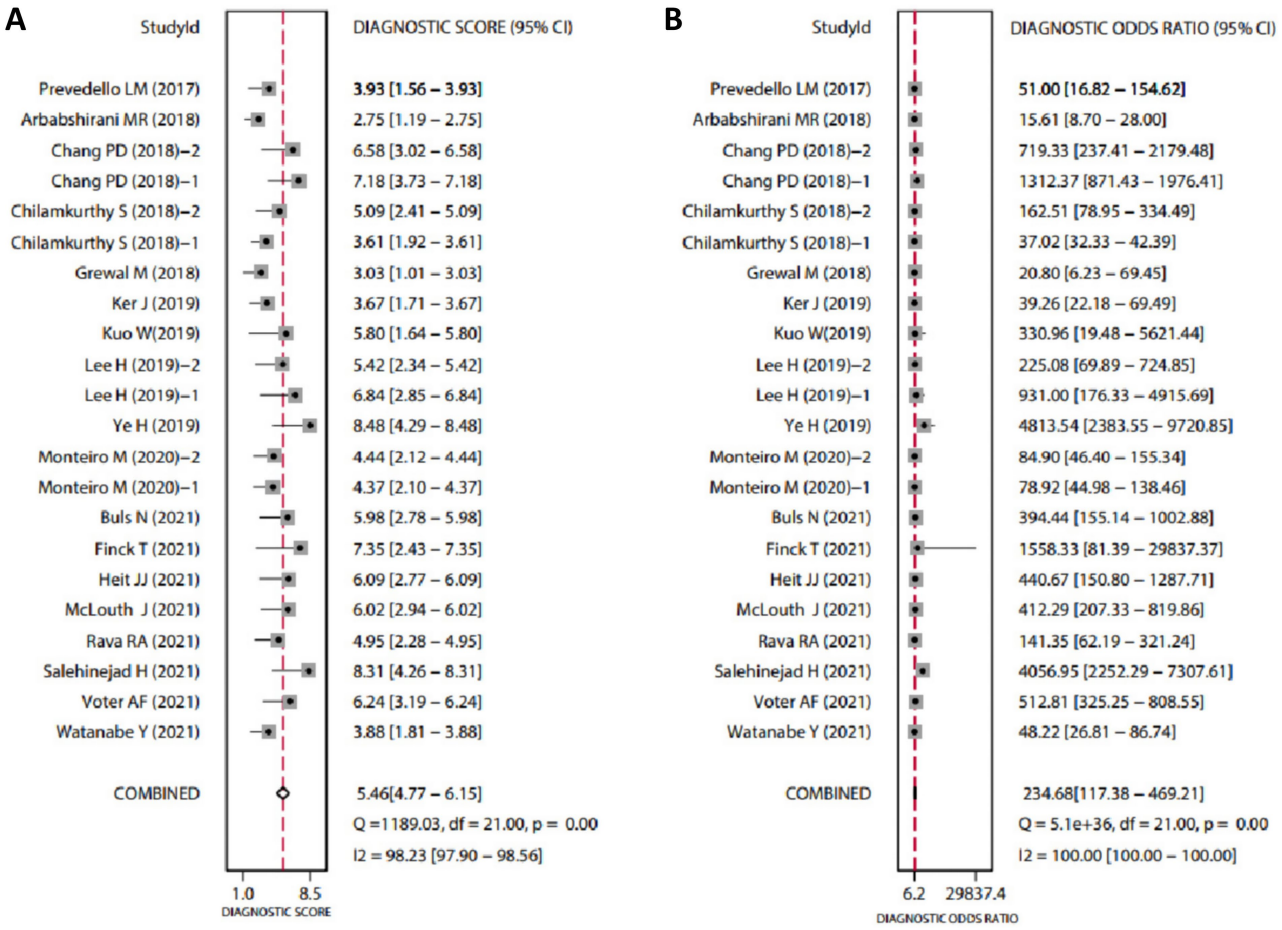
Forest plots of diagnostic scores and odds ratios for AI-assisted CT imaging in diagnosing ICH. (**A, B**) The *Q*-test yielded *p* < 0.05, indicating statistically significant heterogeneity among the included studies. An *I*^2^ value greater than 50% suggests considerable heterogeneity. Higher Diagnostic Scores and DOR indicate greater diagnostic accuracy.

The diagnostic data from the included studies are detailed in Table 1. Based on this data, we conducted a meta-analysis of diagnostic tests using Stata software. The analysis revealed that the SEN of AI-assisted CT imaging for diagnosing ICH was 0.94 (95% CI: 0.90–0.96) (Figure 1A), and the SPE was also 0.94 (95% CI: 0.91–0.96) (Figure 1B). The diagnostic score was 5.46 (95% CI: 4.77–6.15) (Figure 2A), and the DOR was 234.68 (95% CI: 117.38–469.21) (Figure 2B). These results indicate that AI demonstrates high accuracy in the intelligent prediction of ICH using CT imaging. It is important to note that the *Q*-test and *I*^2^ statistic suggest some heterogeneity among the studies, possibly due to differences in experimental design.

**Table 1:** Diagnostic results included in the literature.

First author	*N*	TP	FP	FN	TN
Watanabe Y (2021) (41)	433	211	38	19	165
Voter AF (2021) (42)	3,605	322	27	74	3,182
Salehinejad H (2021) (43)	3,518	1,228	45	15	2,230
Rava RA (2021) (44)	360	245	12	13	90
McLouth J (2021) (45)	814	233	22	14	545
Heit JJ (2021) (46)	308	151	7	7	143
Finck T (2021) (47)	166	137	0	4	25
Buls N (2021) (48)	500	331	20	6	143
Monteiro M (2020)-1 (49)	655	422	47	19	167
Monteiro M (2020)-2 (49)	490	185	20	28	257
Ye H (2019) (50)	2,836	1,799	10	37	990
Lee H (2019)-1 (51)	200	98	5	2	95
Lee H (2019)-2 (51)	196	73	6	6	111
Kuo W (2019) (52)	200	25	0	23	152
Ker J (2019) (53)	424	238	23	34	129
Grewal M (2018) (54)	77	39	9	5	24
Chilamkurthy S (2018)-1 (55)	23,342	2,247	4,099	248	16,748
Chilamkurthy S (2018)-2 (55)	491	194	28	11	258
Chang PD (2018)-1 (56)	11,060	875	254	26	9,905
Chang PD (2018)-2 (56)	764	78	18	4	664
Arbabshirani MR (2018) (57)	350	60	34	26	230
Prevedello LM (2017) (58)	130	45	12	5	68

TP, True Positive; FP, False Positive; FN, False Negative; TN, True Negative.

Additionally, the diagnostic value of the method was comprehensively assessed using the AUC from the SROC curve, as shown in Supplementary Figure S8A. The AUC was 0.98 (95% CI: 0.96–0.99), indicating excellent diagnostic performance. We also constructed a Fagan nomogram to calculate the post-test probability based on a set pre-test probability (Supplementary Figure S8B). Furthermore, Deek’s funnel plot showed no significant publication bias (*p* = 0.88) (Supplementary Figure S8C). Finally, we plotted a scatter distribution (Supplementary Figure S8D) to further evaluate the diagnostic value of the included studies.

In summary, the results demonstrate that AI shows high accuracy in predicting ICH from CT imaging.

### ICH and poor collateral circulation correlate with worse prognosis in IS patients

Previous meta-analyses have demonstrated the effectiveness of AI in predicting ICH. This study aims to predict post-interventional hemorrhage and collateral circulation in patients with acute IS using preoperative one-stop CT scanning, allowing for early preventive measures. We collected data from 58 patients who underwent intervention for acute IS at our hospital between January 2022 and March 2023. All patients underwent preoperative head CT scans, and detailed clinical information was gathered. Patients were grouped based on ICH and collateral circulation. ICH was categorized into two groups: Bleeding and Non-Bleeding. Based on the collateral score, patients were divided into three groups: Good (scores 3–4), Partial (score 2), and Poor (scores 0–1).

Our findings revealed that patients in the Bleeding group had significantly lower collateral scores compared to the Non-Bleeding group (*p* = 0.049). Additionally, the NIHSS (*p* = 0.017) and mRS (*p* = 0.019) scores were significantly higher in the Poor collateral circulation group compared to the Good group. The incidence of subarachnoid hemorrhage (SAH) was also significantly higher in the Poor group (*p* = 0.019) (Table 2). These results indicate a strong association between collateral circulation and ICH, both of which are linked to patient prognosis.

**Table 2:** Baseline characteristics of 58 patients with ischemic stroke.

Characteristics	Intracranial hemorrhage		*P* value	Collateral circulation			*P* value
	Bleeding (*n* = 35)	Non-bleeding (*n* = 23)		Good (*n* = 8)	Partial (*n* = 12)	Poor (*n* = 36)	
Age, mean ± sd	67.6 ± 13.916	67.478 ± 13.18	0.974	64.125 ± 19.052	72.333 ± 12.01	67 ± 12.913	0.373
Gender, *n* (%)			0.879				0.81
Male	25 (43.1%)	16 (27.6%)		6 (10.7%)	9 (16.1%)	24 (42.9%)	
Female	10 (17.2%)	7 (12.1%)		2 (3.6%)	3 (5.4%)	12 (21.4%)	
History of diabetes, *n* (%)	7 (12.1%)	6 (10.3%)	0.587	2 (3.6%)	3 (5.4%)	7 (12.5%)	0.889
History of hypertension, *n* (%)	21 (36.2%)	15 (25.9%)	0.689	5 (8.9%)	6 (10.7%)	24 (42.9%)	0.587
History of hyperlipidemia, *n* (%)	10 (17.2%)	6 (10.3%)	0.836	2 (3.6%)	3 (5.4%)	11 (19.6%)	0.907
History of coronary heart disease, *n* (%)	9 (15.5%)	6 (10.3%)	0.975	1 (1.8%)	3 (5.4%)	10 (17.9%)	0.665
History of atrial fibrillation, *n* (%)	11 (19%)	7 (12.1%)	0.936	0 (0%)	4 (7.1%)	13 (23.2%)	0.129
History of stroke, *n* (%)	5 (8.6%)	4 (6.9%)	1	1 (1.8%)	2 (3.6%)	6 (10.7%)	0.957
History of antiplatelets, *n* (%)	4 (6.9%)	0 (0%)	0.25	1 (1.8%)	2 (3.6%)	1 (1.8%)	0.221
History of anticoagulant drugs, *n* (%)	3 (5.2%)	1 (1.7%)	0.927	0 (0%)	1 (1.8%)	3 (5.4%)	0.698
History of statins, *n* (%)	4 (6.9%)	5 (8.6%)	0.49	2 (3.6%)	1 (1.8%)	6 (10.7%)	0.602
Baseline NIHSS score, mean ± sd	13.429 ± 6.041	11.091 ± 5.723	0.152	7.375 ± 3.543	12.833 ± 7.184	13.833 ± 5.294	0.017
Baseline mRS score, median (IQR)	4 (4, 5)	4.5 (2.5, 5)	0.65	3 (0.75, 4)	5 (4, 5)	5 (4, 5)	0.019
CT-aspects score, median (IQR)	10 (9.25, 10)	10 (10, 10)	0.224	10 (9.5, 10)	10 (10, 10)	10 (10, 10)	0.608
Baseline TICI, *n* (%)			0.725				0.246
0	28 (49.1%)	18 (31.6%)		5 (8.9%)	9 (16.1%)	32 (57.1%)	
1	1 (1.8%)	0 (0%)		0 (0%)	0 (0%)	1 (1.8%)	
2	6 (10.5%)	4 (7%)		3 (5.4%)	3 (5.4%)	3 (5.4%)	
Postoperative TICI, *n* (%)			0.088				0.103
0	2 (3.5%)	0 (0%)		0 (0%)	2 (3.6%)	0 (0%)	
2	14 (24.6%)	4 (7%)		3 (5.4%)	3 (5.4%)	12 (21.4%)	
3	19 (33.3%)	18 (31.6%)		5 (8.9%)	7 (12.5%)	24 (42.9%)	
Bleeding							
rPH, *n* (%)	3 (5.2%)			0 (0%)	0 (0%)	3 (5.4%)	0.415
IVH, *n* (%)	5 (8.6%)			0 (0%)	0 (0%)	5 (8.9%)	0.218
SAH, *n* (%)	24 (41.4%)			0 (0%)	4 (7.1%)	19 (33.9%)	0.019
ICH, mean ± sd	2.5429 ± 1.221			0 (0, 0.25)	2 (0, 3)	1 (0, 3.25)	0.143
Total bleeding, *n* (%)				2 (3.6%)	8 (14.3%)	24 (42.9%)	0.082
Collateral score, median (IQR)	1 (1, 2)	1 (1, 2.75)	0.049				

NIHSS, National Institutes of Health Stroke Scale; mRS, modified Rankin Scale; TICI, thrombolysis in cerebral infarction; rPH, remote parenchymal hemorrhage; IVH, intraventricular hemorrhage; SAH, subarachnoid hemorrhage; ICH, Symptomatic intracranial hemorrhage.

Previous studies have shown that stroke patients with good collateral circulation have smaller infarct volumes and better clinical outcomes (26, 27). A systematic review and meta-analysis further demonstrated that patients who experience ICH during the acute phase of IS, especially those undergoing endovascular therapy, face a significantly increased risk of poor prognosis compared to those without hemorrhage (28). Therefore, accurate prediction of post-interventional ICH and collateral circulation is crucial for improving the prognosis of IS patients.

### Construction and testing of a deep machine learning data analysis environment

Based on the results of the previous clinical data analysis, we further identified that ICH and collateral circulation significantly impact patient prognosis. To address the challenge of predicting ICH and collateral circulation in IS patients, we developed several deep machine-learning models aimed at selecting accurate and stable diagnostic tools. The training dataset consisted of CT imaging data from 58 IS patients treated at our hospital, where ICH was classified into hemorrhage and non-hemorrhage categories, and collateral circulation was categorized as good, partial, or poor. For the assessment of ICH, the CQ500 dataset was used as the test set. In contrast, for collateral circulation evaluation, 20% of the CT imaging data from the 58 IS patients were randomly selected as an independent test set, with the remaining data used for model training.

Regarding pre-training parameter settings, network weights were initialized based on pre-training results, and the custom score threshold was set at 0.5. The number of iterations was initially set to 50, with the option to continue training based on test results to further converge the loss value. The batch processing parameter was set to 12, depending on the graphics card used, to prevent memory overflow. The stride was set to 10, and padding was set to 1. The detailed parameter settings are shown in Table 3.

**Table 3:** Parameter settings for machine learning data analysis.

Parameter name	Parameter value
Weight	Initial weight
Learning rate	0.001
Iterations	50
Batch processing capacity	12
Step	10
Padding	1

Supplementary Figure S9 presents the confusion matrices for the classification results of the training set using five deep machine learning models (Densenet169, InceptionResNetV2, InceptionV4, MobileNetV3Small, SEResNet101). In the confusion matrices, the vertical axis represents the true labels, while the horizontal axis represents the predicted labels. Generally, the darker the diagonal line in the confusion matrix, the higher the classification accuracy. Analyzing the confusion matrices, we observe that in the classification of bleeding versus non-bleeding, the models InceptionResNetV2 (Bleeding: 0.91; Non-Bleeding: 0.97), InceptionV4 (Bleeding: 0.97; Non-Bleeding: 0.98), and SEResNet101 (Bleeding: 1.00; Non-Bleeding: 0.99) achieved higher classification accuracy. Similarly, in the three-class classification of collateral circulation, the models InceptionV4 (good: 0.87; partial: 0.92; poor: 0.97) and SEResNet101 (good: 0.89; partial: 0.98; poor: 0.99) also demonstrated higher classification accuracy.

### Comparison of the diagnostic accuracy of five DL models for ICH in post-intervention IS patients

**Figure 3:**
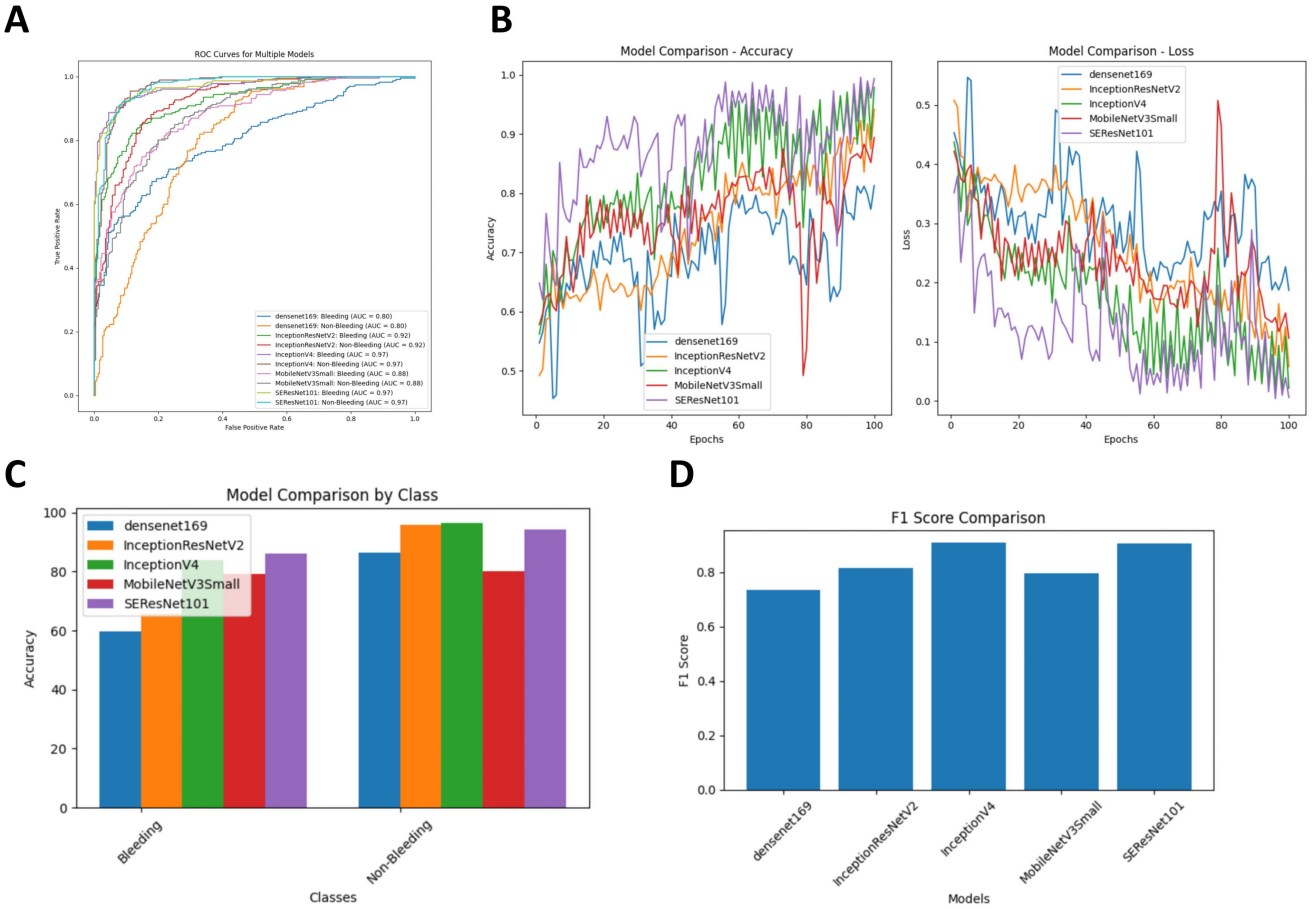
Comparison of diagnostic accuracy for ICH following intervention across five DL algorithms. **(A)** Comparison of ROC curves for the five DL algorithms; **(B)** Training and cross-validation loss during the training period for the five DL algorithms; **(C)** Comparison of diagnostic accuracy for hemorrhage versus non-hemorrhage cases among the five DL algorithms; **(D)** Comparison of F1 Scores for the five DL algorithms.

To evaluate the effectiveness of the classification models, we calculated four performance metrics: classification accuracy (ACC), SEN, SPE, and area under the receiver operating characteristic curve (AUC). As shown in Table 4, InceptionV4 and SEResNet101 significantly outperformed the other three models. For the diagnosis of hemorrhage, their ACC values were 83.98 and 86.15%, respectively, and for the diagnosis of non-hemorrhage cases, their ACC values were 96.53 and 94.10%, respectively. In the binary classification of hemorrhage, both InceptionV4 and SEResNet101 achieved an AUC as high as 97.00% (Figure 3).

**Table 4:** Comparison of diagnostic accuracy of five deep machine learning algorithms for post-interventional intracranial hemorrhage.

Intracranial hemorrhage	Bleeding (%)				Non-Bleeding (%)			
	ACC	SEN	SPE	AUC	ACC	SEN	SPE	AUC
Densenet169	59.74	58.44	87.15	80	86.46	87.15	58.44	80
InceptionResNetV2	65.8	68.83	94.79	92	95.83	94.79	68.83	92
InceptionV4	83.98	76.62	95.48	97	96.53	95.48	76.62	97
MobileNetV3Small	79.22	76.78	78.12	88	80.21	78.21	76.78	88
SEResNet101	86.15	87.87	95.83	97	94.1	95.83	87.87	97

ACC, accuracy; SEN, sensitivity; SPE, specificity; AUC, area under the operating characteristic curve.

**Figure 4:**
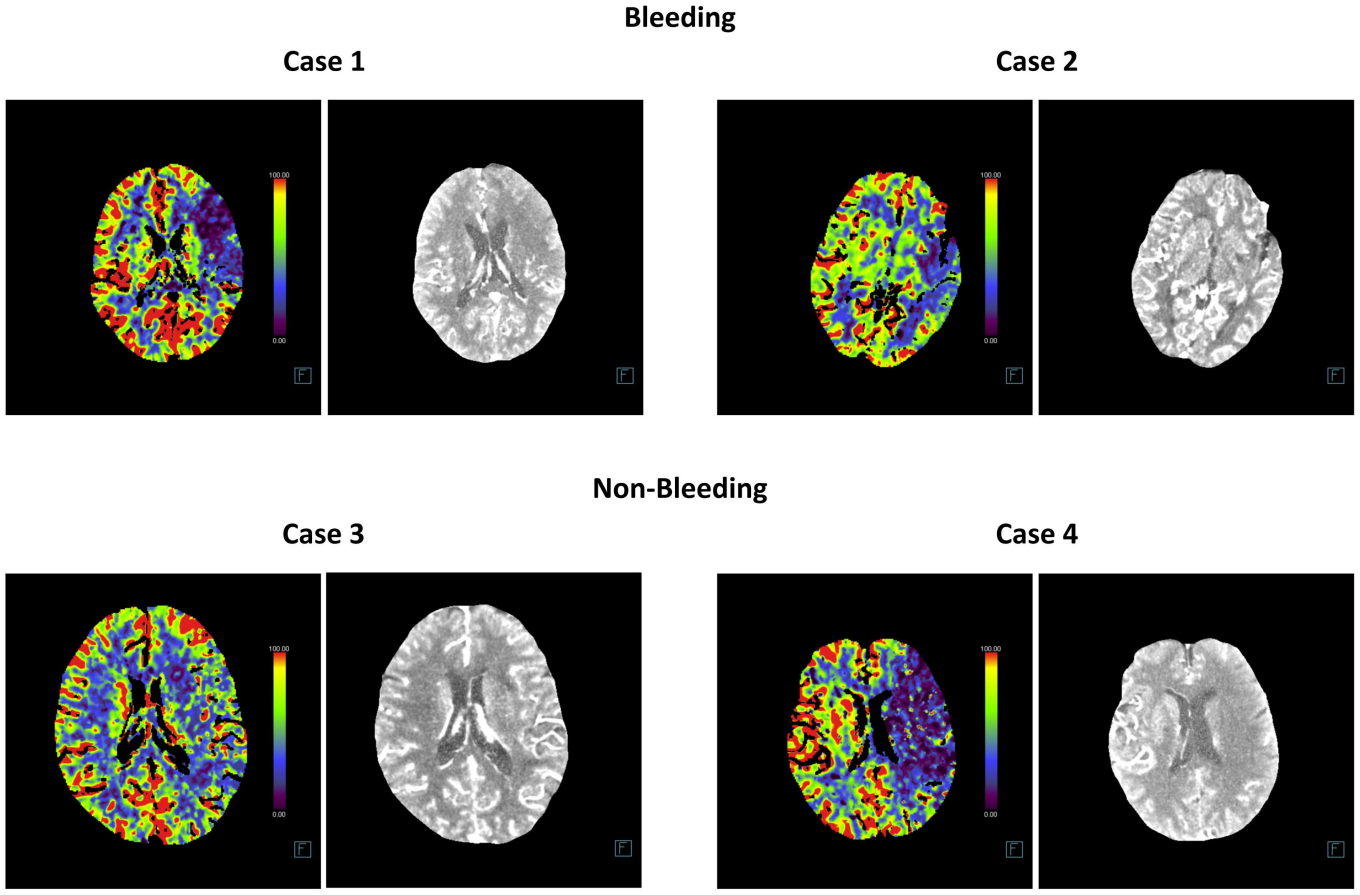
Representative head CT slices for diagnosing post-interventional ICH using InceptionV4 and SEResNet101 algorithms. The left image in each case represents the heatmap of the CT scan used for DL, while the right image shows the original CT slice.

Additionally, we compared the training and cross-validation losses of the five DL models during the training phase and ranked them based on accuracy and F1 score. The results consistently indicate that the InceptionV4 and SEResNet101 models exhibit higher accuracy in the intelligent diagnosis of ICH in post-intervention IS patients, followed by InceptionResNetV2, MobileNetV3Small, and Densenet169 (Figures 3B–D). Furthermore, we present TP examples of ICH diagnoses made by the InceptionV4 and SEResNet101 models (Figure 4).

### Comparison of the diagnostic accuracy of five deep machine learning models for collateral circulation in post-intervention IS patients

**Figure 5:**
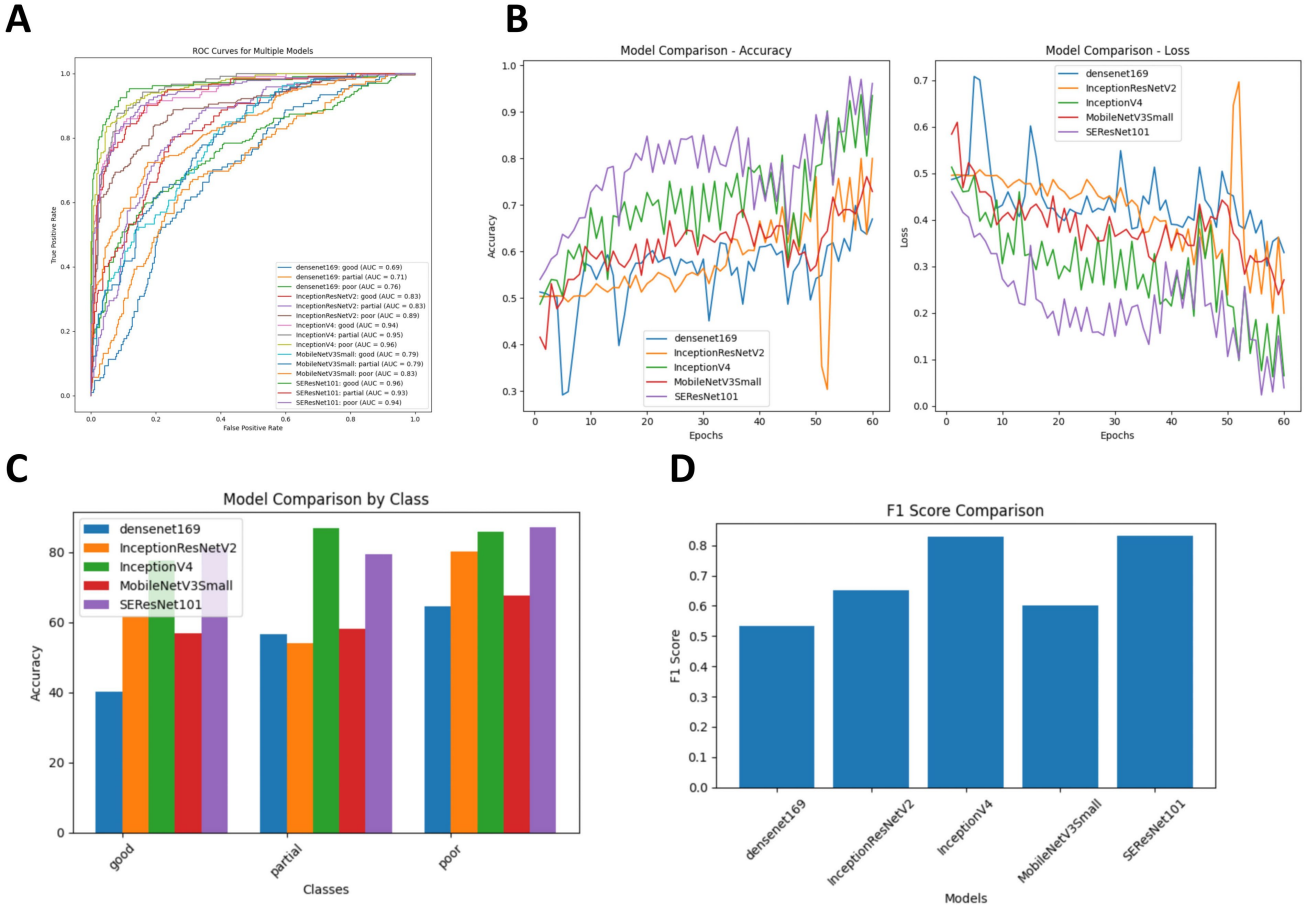
Comparison of diagnostic accuracy for post-interventional collateral circulation among five DL models. **(A)** Comparison of ROC curves for the five DL models; **(B)** Training and cross-validation loss during the training phase for the five models; **(C)** Accuracy comparison in the diagnosis of Collateral Circulation; **(D)** F1 Score comparison for the five DL models.

**Figure 6:**
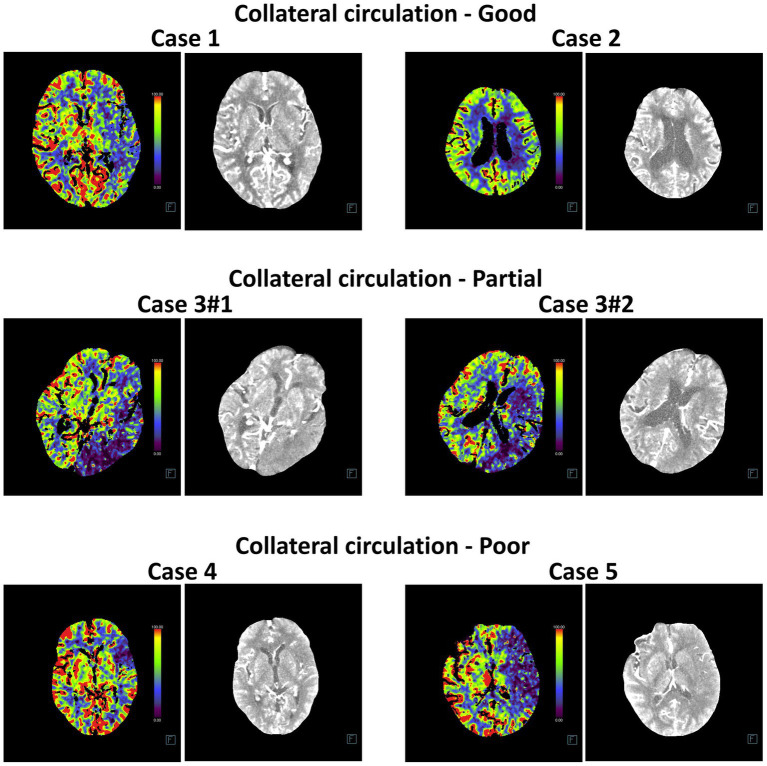
Representative head CT slices for collateral circulation diagnosis using InceptionV4 and SEResNet101 algorithms. The left image in each case represents the heatmap of the CT scan used for DL, while the right image shows the original CT slice.

In addition, we compared the diagnostic accuracy of five deep machine learning models for evaluating collateral circulation in post-intervention IS patients. As shown in Table 5, for the Partial vs. Good diagnosis, the ACC values of InceptionV4 and SEResNet101 were 82.23 and 80.41%, respectively, with AUCs reaching 94.50%. For the Poor vs. Good diagnosis, the ACC values were 81.67 and 84.19%, respectively, with AUCs reaching 95.00%. These results demonstrate that InceptionV4 and SEResNet101 significantly outperformed the other three models (Figures 5A–D). Examples of TP cases diagnosed by the InceptionV4 and SEResNet101 models are presented in Figure 6. These findings suggest that the InceptionV4 and SEResNet101 models exhibit high accuracy in the intelligent diagnosis of collateral circulation in post-intervention IS patients.

**Table 5:** Comparison of diagnostic accuracy of different algorithm models for post-interventional collateral circulation.

Intracranial hemorrhage	Bleeding (%)				Non-bleeding (%)			
	ACC	SEN	SPE	AUC	ACC	SEN	SPE	AUC
Densenet169	48.37	48.37	79.03	70	52.42	52.42	78.74	72.5
InceptionResNetV2	57.89	57.89	85.19	83	70.92	70.92	82.65	86
InceptionV4	82.23	82.23	92.18	94.5	81.67	81.67	92.6	95
MobileNetV3Small	57.6	57.6	80.83	79	62.34	62.34	81.31	81
SEResNet101	80.41	80.41	93.87	94.5	84.19	84.19	91.38	95

ACC, accuracy; SEN, sensitivity, SPE, specificity; AUC, area under the operating characteristic curve.

### Kdr, Lcn2, and Pxn as key biomarkers of ICH and poor collateral circulation following reperfusion in the MCAO mouse model

**Figure 7:**
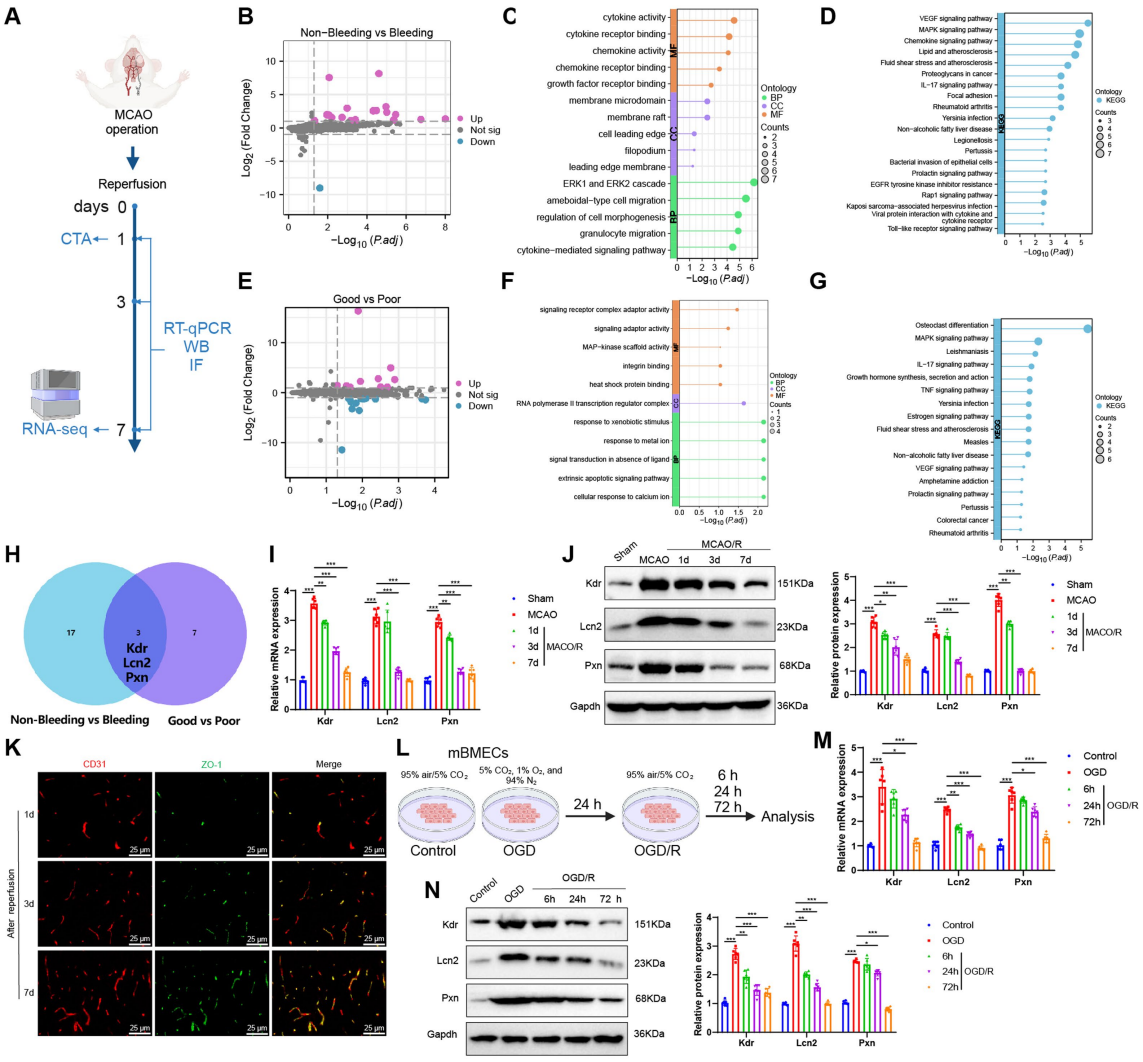
Transcriptomic features of ICH and poor collateral circulation in MCAO model mice post-reperfusion. **(A)** Flowchart of animal experiments; **(B)** Volcano plot showing differential analysis between Non-Bleeding group (*n* = 3) and Bleeding group (*n* = 3); **(C,D)** GO **(C)** and KEGG **(D)** enrichment analysis of DEGs between Non-Bleeding and Bleeding groups, shown as lollipop plots; **(E)** Volcano plot showing differential analysis between Good group (*n* = 3) and Poor group (*n* = 3); **(F,G)** GO **(F)** and KEGG **(G)** enrichment analysis of DEGs between Good and Poor groups, shown as lollipop plots; **(H)** Venn diagram of upregulated genes intersected between the two groups; **(I,J)** RT-qPCR **(I)** and WB **(J)** analysis of Kdr, Lcn2, and Pxn expression levels in brain tissues of each group; **(K)** Immunofluorescence staining detecting CD31 and ZO-1 positive expression in brain tissues of mice, Scale bar = 100 μm; **(L)** Flowchart of cell experiments; **(M,N)** RT-qPCR **(M)** and WB **(N)** analysis of Kdr, Lcn2, and Pxn expression levels in cells from each group; * indicates *p* < 0.05, ** indicates *p* < 0.01, *** indicates *p* < 0.001. Each group in animal experiments consisted of 6 mice, and cell experiments were conducted in triplicate.

To further explore the transcriptomic characteristics associated with ICH and poor collateral circulation following IS reperfusion, we established a MCAO mouse model. On the first day after reperfusion, mice were categorized based on the presence of ICH, determined via CTA, into Non-Bleeding and Bleeding groups. Additionally, mice were classified according to the status of their collateral circulation into Good and Poor groups. For each group, brain tissue samples from the ischemic penumbra were collected for Transcriptome Sequencing (RNA-seq) (Figure 7A).

We first compared the Non-Bleeding and Bleeding groups, using a threshold of |logFC| > 1 and *p* < 0.05, and identified 20 significantly upregulated and 1 significantly downregulated gene (Figure 7B). GO and KEGG enrichment analyses revealed that these DEGs were primarily involved in BP such as the ERK1 and ERK2 cascade, ameboidal-type cell migration, granulocyte migration, regulation of cell morphogenesis, and cytokine-mediated signaling pathways. They were also enriched in CC, including membrane rafts, membrane microdomains, filopodia, cell leading edges, and leading-edge membranes. Furthermore, MF such as cytokine activity, cytokine receptor binding, chemokine activity, chemokine receptor binding, and growth factor receptor binding were highlighted, along with pathways like VEGF, MAPK, cytokine, and IL-17 signaling (Figures 7C,D).

We then conducted a differential expression analysis between the Good and Poor groups. Using a threshold of |logFC| > 1 and *p* < 0.05, we identified 10 significantly upregulated genes and 13 significantly downregulated genes (Figure 7E). GO and KEGG enrichment analyses revealed that these DEGs were primarily enriched in BP, such as cellular response to calcium ions, extrinsic apoptotic signaling pathways, signal transduction in the absence of a ligand, response to metal ions, and response to xenobiotic stimuli. They were also enriched in CC such as the RNA polymerase II transcription regulator complex, and MF including signaling receptor complex adaptor activity, signaling adaptor activity, heat shock protein binding, integrin binding, and MAP-kinase scaffold activity, as well as signaling pathways like MAPK, IL-17, TNF, estrogen, and VEGF (Figures 7F,G).

Finally, to further explore the shared transcriptomic characteristics between ICH and poor collateral circulation, we intersected the DEGs from both comparisons and identified three common genes: Kdr, Lcn2, and Pxn, all of which were upregulated; no downregulated genes were found in the intersection (Figure 7H). Subsequently, we collected brain tissue samples from the Sham group, MCAO group, and mice with favorable outcomes after reperfusion (i.e., no ICH and good collateral circulation). We then assessed the expression changes of Kdr, Lcn2, and Pxn using RT-qPCR and Western Blot (WB). The results showed that compared to the Sham group, both mRNA and protein levels of Kdr, Lcn2, and Pxn were significantly upregulated in the MCAO group. Furthermore, their expression levels were significantly downregulated after reperfusion and gradually decreased over time (Figures 7I,J), which is consistent with the results from the RNA-seq.

CD31 is a commonly used marker for endothelial cells and is employed to assess angiogenesis, while ZO-1 is a tight junction protein indicative of blood–brain barrier (BBB) integrity. Therefore, we performed immunofluorescence staining to assess the expression of CD31 and ZO-1 in brain tissue samples of mice at 1, 3, and 7 days post-reperfusion. This allowed us to comprehensively evaluate the processes of angiogenesis and BBB damage and repair following IS. We observed a gradual increase in the fluorescence intensity of CD31 and ZO-1 over time (Figure 7K), indicating enhanced angiogenesis and ongoing BBB repair. This also suggests that the expression changes of Kdr, Lcn2, and Pxn are consistent with the processes of angiogenesis and BBB restoration.

Subsequently, we examined the expression changes of Kdr, Lcn2, and Pxn in mBMECs under normoxic conditions (Control group) and after OGD or OGD/R treatment (Figure 7L). Compared to the Control group, the OGD group exhibited a significant upregulation in the mRNA and protein expression levels of Kdr, Lcn2, and Pxn. However, after OGD/R treatment, the expression levels of these three markers were significantly downregulated compared to the OGD group and continued to decrease over time (Figures 7M,N). These findings are consistent with the results of our *in vivo* experiments.

In summary, we propose that the dynamic expression changes of Kdr, Lcn2, and Pxn following IS reperfusion could provide an additional reference value for AI-assisted CT diagnosis.

## Discussion

In the treatment of IS, the occurrence of ICH following interventional procedures and the status of collateral circulation significantly impact patient prognosis. ICH is a severe complication that can exacerbate the patient’s condition, increasing both mortality and disability rates (5, 6). Early identification and prediction of ICH are crucial for timely intervention, making accurate post-procedural prediction of ICH a major challenge for clinicians (29). Concurrently, collateral circulation, an essential indicator of CBF compensation, plays a critical role in patient recovery. Robust collateral circulation can enhance blood supply to ischemic regions, promoting brain tissue recovery, whereas poor collateral circulation is associated with worse clinical outcomes (30).

In this study, we selected 18 articles, encompassing 22 datasets, for a meta-analysis. The results indicate that AI demonstrates a high accuracy in predicting ICH from CT images, confirming its effectiveness in medical imaging analysis. Notably, AI shows significant potential in the rapid and accurate diagnosis of ICH. This meta-analysis provides robust data supporting the future integration of AI technologies into clinical diagnostics, highlighting their critical value in enhancing diagnostic efficiency and accuracy. Consequently, in our subsequent work, we applied five DL models—Densenet169, InceptionResNetV2, InceptionV4, MobileNetV3Small, and SEResNet101—to predict post-interventional ICH and collateral circulation, achieving notable results.

The application of DL models in medical imaging analysis signifies a transition from traditional diagnostic methods to more advanced technologies. These models are distinguished by their ability to process large, complex datasets and extract critical features, a task unattainable by traditional diagnostic methods (31, 32). For instance, InceptionV4 can consider multiple layers and perspectives when processing image data (31), while SEResNet101 excels in feature recognition (33). Compared to commonly used models in previous studies, such as basic CNN or standard machine learning algorithms, these models offer significant advantages (22, 34).

**Figure 8:**
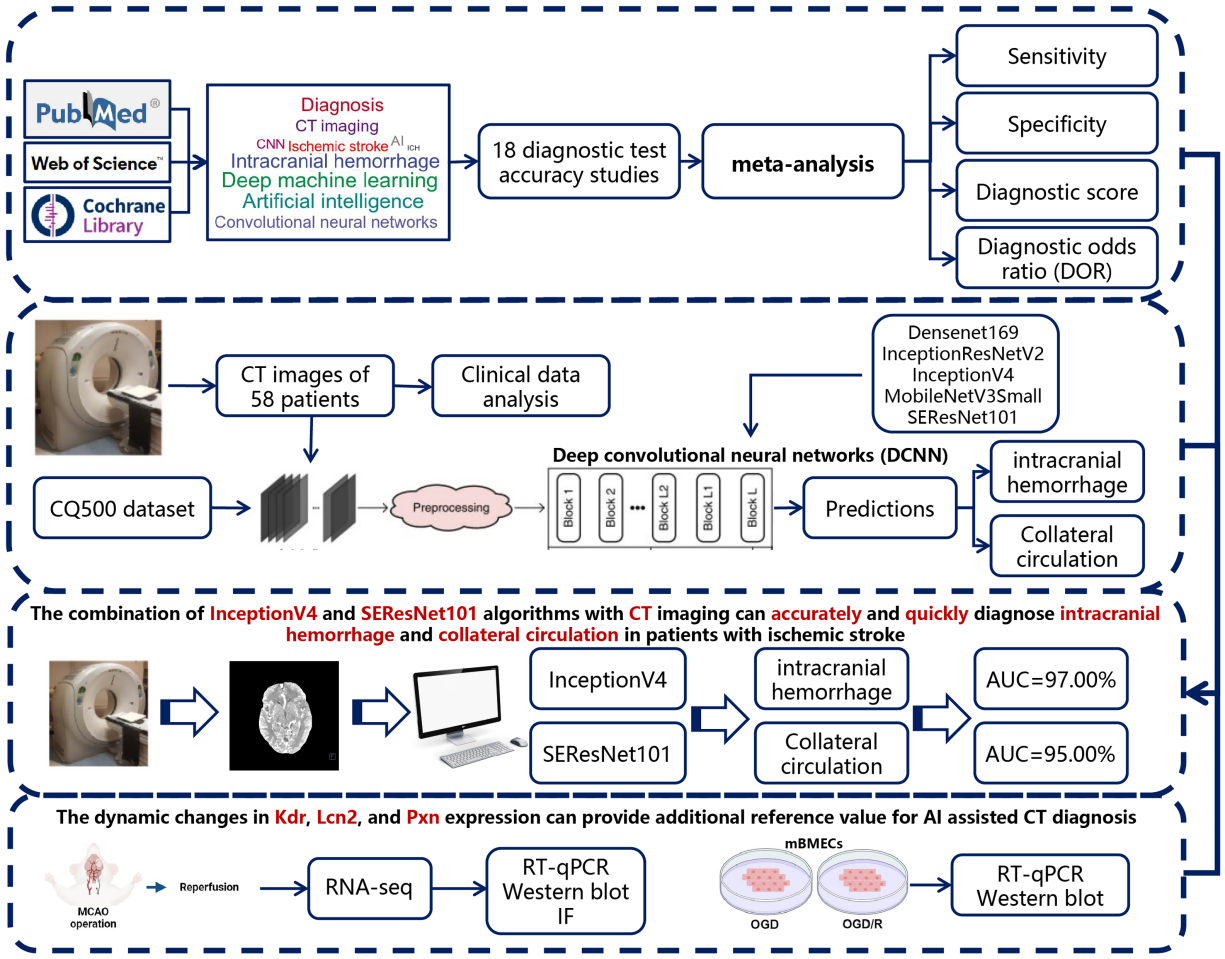
Overview of the study’s analytical workflow and main conclusions.

The dataset used in this study consists of preoperative head CT scans from 58 patients who underwent interventional treatment for acute IS, providing a unique perspective and depth to our research. In evaluating algorithm performance, our study demonstrates that InceptionV4 and SEResNet101 achieved unprecedented levels of predictive accuracy, particularly in the binary classification of ICH, with both algorithms surpassing 95% in AUC scores. This high level of performance not only underscores the effectiveness of DL models in medical imaging analysis but also highlights their robust capability in handling highly complex and variable medical data (35–37). Based on the meta-analysis, clinical data analysis, and deep machine learning results, we conclude that the combination of InceptionV4 or SEResNet101 with preoperative CT imaging can accurately and rapidly predict post-intervention ICH and collateral circulation in IS patients, providing critical evidence for timely treatment. Additionally, through RNA-seq and *in vivo* and *in vitro* experiments, we identified key biomarkers related to poor ICH and collateral circulation outcomes following reperfusion in a MCAO mouse model. The dynamic expression changes of Kdr, Lcn2, and Pxn offer additional reference value for AI-assisted CT diagnosis (Figure 8).

Regarding the clinical application potential of these three biomarkers, we believe their utility should be distinguished based on their biodistribution and detectability. Lcn2 is a secreted protein, and previous studies have shown that it can be detected in the bloodstream, suggesting its potential as a peripheral blood biomarker (38). In contrast, Kdr (VEGFR2) and Pxn (Paxillin) are primarily membrane-associated or cytoskeletal proteins, typically confined to tissue or cellular expression, making it difficult to monitor their dynamic changes through routine blood tests (39, 40). Therefore, in clinical practice, Lcn2 is more suitable as a candidate biomarker for liquid biopsy to assist in early risk assessment. Meanwhile, Kdr and Pxn are currently more appropriate as tissue-level references or targets in transcriptomic studies associated with imaging. Future research should focus on developing non-invasive imaging tracers or metabolic monitoring techniques to expand their clinical applicability. Additionally, we recommend further exploration of the potential correlations between these biomarkers and radiomic features. For instance, integrating biomarker expression patterns with CT imaging characteristics may facilitate the development of combined image-molecular predictive models. This integrative approach could bridge the gap between CT imaging, AI-based prediction, and molecular mechanism interpretation, thereby enhancing the interpretability and credibility of AI models in clinical settings.

Despite the significant progress achieved in this study, several limitations remain. First, the relatively small sample size may limit the generalizability of our findings. Additionally, although the algorithms employed in this study performed well on the current dataset, their effectiveness in different datasets or clinical settings requires further validation. Future studies should, therefore, aim to test these findings in larger samples and more diverse clinical environments. Although our model demonstrated robust performance on both internal and public datasets, it has not yet been validated using independent external datasets. This may limit its generalizability across diverse patient populations and imaging settings. Future research will focus on conducting multicenter, prospective studies with external validation cohorts to further confirm the model’s reliability and clinical applicability in real-world scenarios. While this study proposed the potential of integrating imaging features and molecular biomarkers to improve predictive accuracy and model interpretability, such integration has not yet been fully achieved. Future studies should explore the construction of multimodal predictive models by correlating radiomic features from preoperative CT images with tissue or serum biomarker expression patterns. This strategy may enhance the biological interpretability and clinical utility of AI-based prediction systems.

Compared to previous research, a notable feature of this study is its in-depth exploration of DL models in medical imaging analysis. While prior studies have primarily focused on traditional machine learning techniques or single DL models, our research combines multiple advanced models and provides a comprehensive evaluation of their performance. Furthermore, our study places particular emphasis on the application of these models in clinical practice, a focus that is less commonly addressed in existing literature.

Overall, this study presents an effective approach that integrates Evidence-Based Medicine (EBM), radiomics, and deep machine learning technologies. It demonstrates the high efficiency and accuracy of the InceptionV4 and SEResNet101 algorithms in predicting post-interventional ICH and collateral circulation in IS patients. Additionally, important biomarkers were identified through RNA-seq and *in vivo* and *in vitro* experiments. These findings not only offer new insights into medical imaging analysis but also provide valuable tools for clinical practice. Despite the limitations, this study lays a solid foundation for future research in this field. Future studies should focus on expanding sample sizes, exploring applications in different clinical contexts, and further optimizing and validating these models. Through these efforts, we can aim to enhance IS treatment outcomes and improve patient prognosis.

## Data Availability

The raw data supporting the conclusions of this article will be made available by the authors, without undue reservation.

## Glossary

ACC: Classification accuracy
AI: Artificial intelligence
ANOVA: Analysis of variance
AUC: Area under the curve
BBB: Blood–brain barrier
CBF: Cerebral BLOOD FLOW
cDNA: Complementary DNA
CT: Computed tomography
CTA: CT angiography
DEGs: Differentially expressed genes
DL: Deep learning
DOR: Diagnostic odds ratio
DSA: Digital subtraction angiography
EBM: Evidence-based medicine
FBS: Fetal bovine serum
FN: False negative
FP: False positive
GO: Gene ontology
HI: Hemorrhagic infarction
ICA: Internal carotid artery
ICH: Intracranial hemorrhage
IS: Ischemic stroke
mBMECs: Mouse brain microvascular endothelial cells
MCA: Middle cerebral artery
MCAO: Middle cerebral artery occlusion
MNI: Montreal neurological institute
mRS: Modified Rankin scale
NLR: Negative likelihood ratio
NIHSS: National institutes of health stroke scale
OGD: Oxygen–glucose deprivation
OGD/R: Oxygen–glucose deprivation/reoxygenation
PLR: Positive likelihood ratio
PH: Parenchymal hemorrhage
RNA-seq: Transcriptome sequencing
RT-qPCR: Real-time quantitative polymerase chain reaction
ROC: Receiver operating characteristic
SEN: Sensitivity
SPE: Specificity
SPM: Statistical parametric mapping
SENet: Squeeze-and-excitation networks
TICI: Thrombolysis in cerebral infarction
TN: True negative
TP: True positive
WB: Western blot
