# Supplementary figures and images for: InceptionV4 and SEResNet101: precise predictors of intracranial hemorrhage and collateral circulation post—ischemic stroke intervention
Source: Front Neurol. 2025 Sep 17;16:1617626. doi: 10.3389/fneur.2025.1617626 (PMC12516262; doi:10.3389/fneur.2025.1617626)

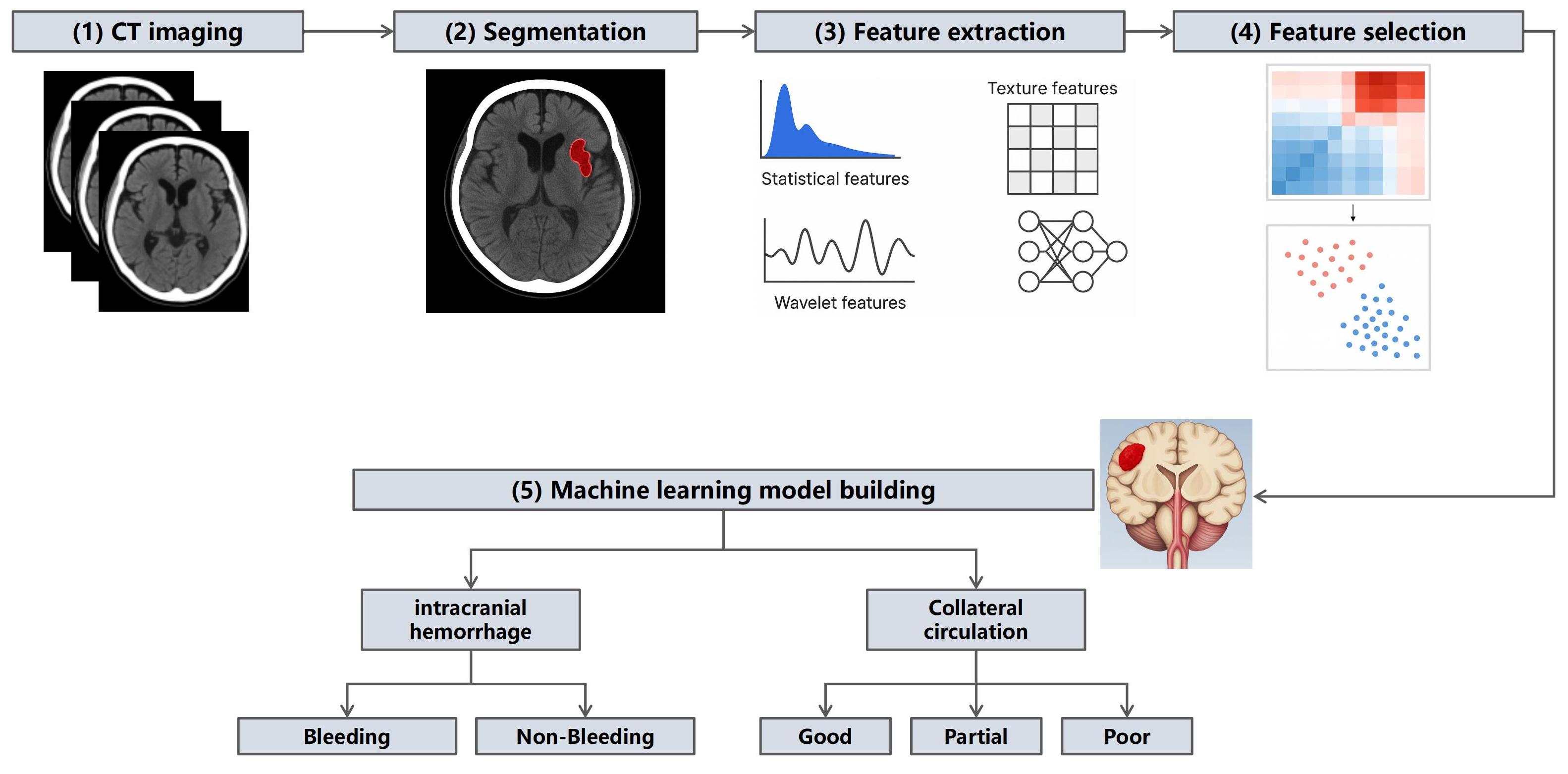

Supplement: Supplementary file 1 [file Image_1.jpg]

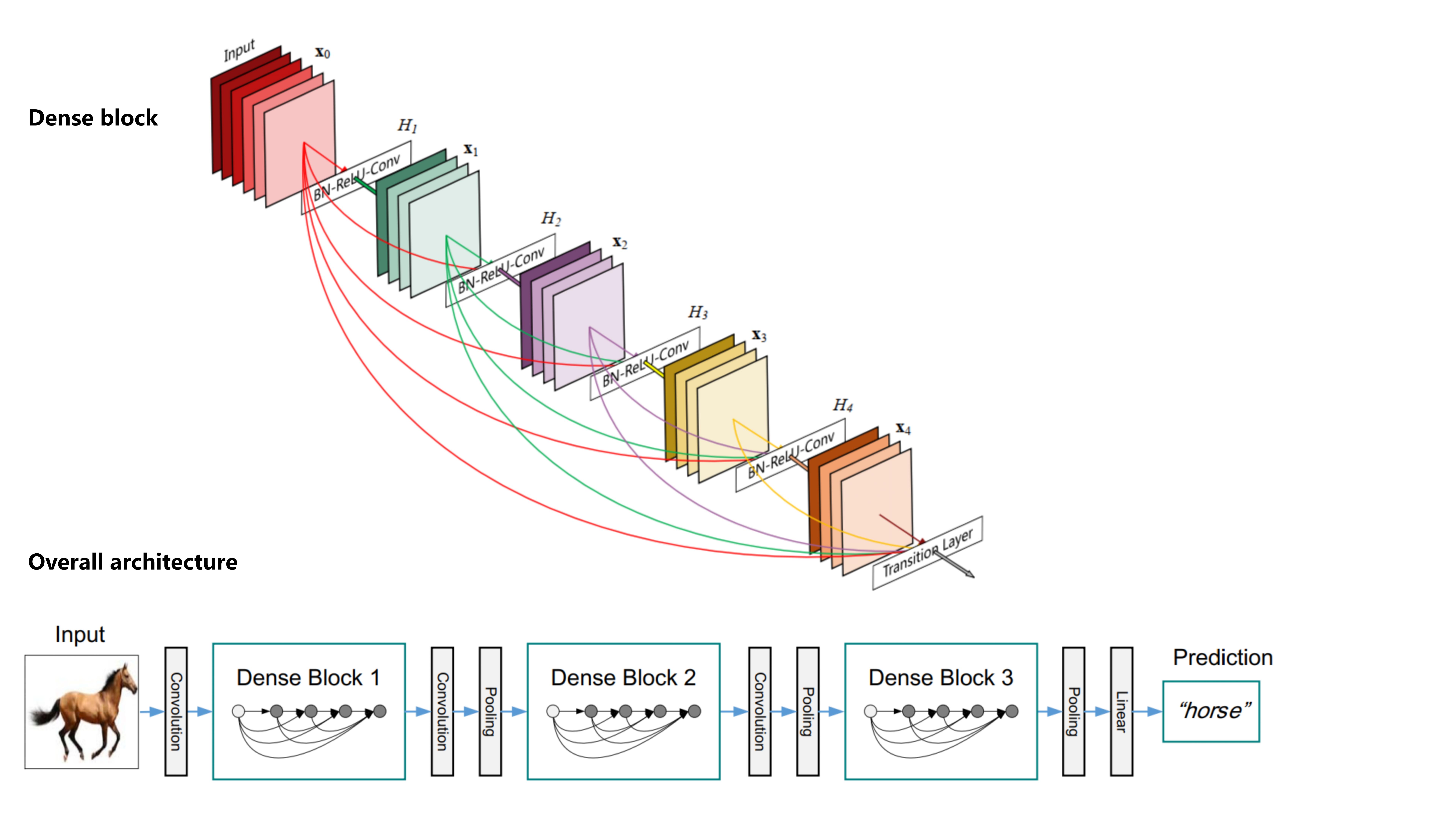

Supplement: Supplementary file 2 [file Image_2.jpeg]

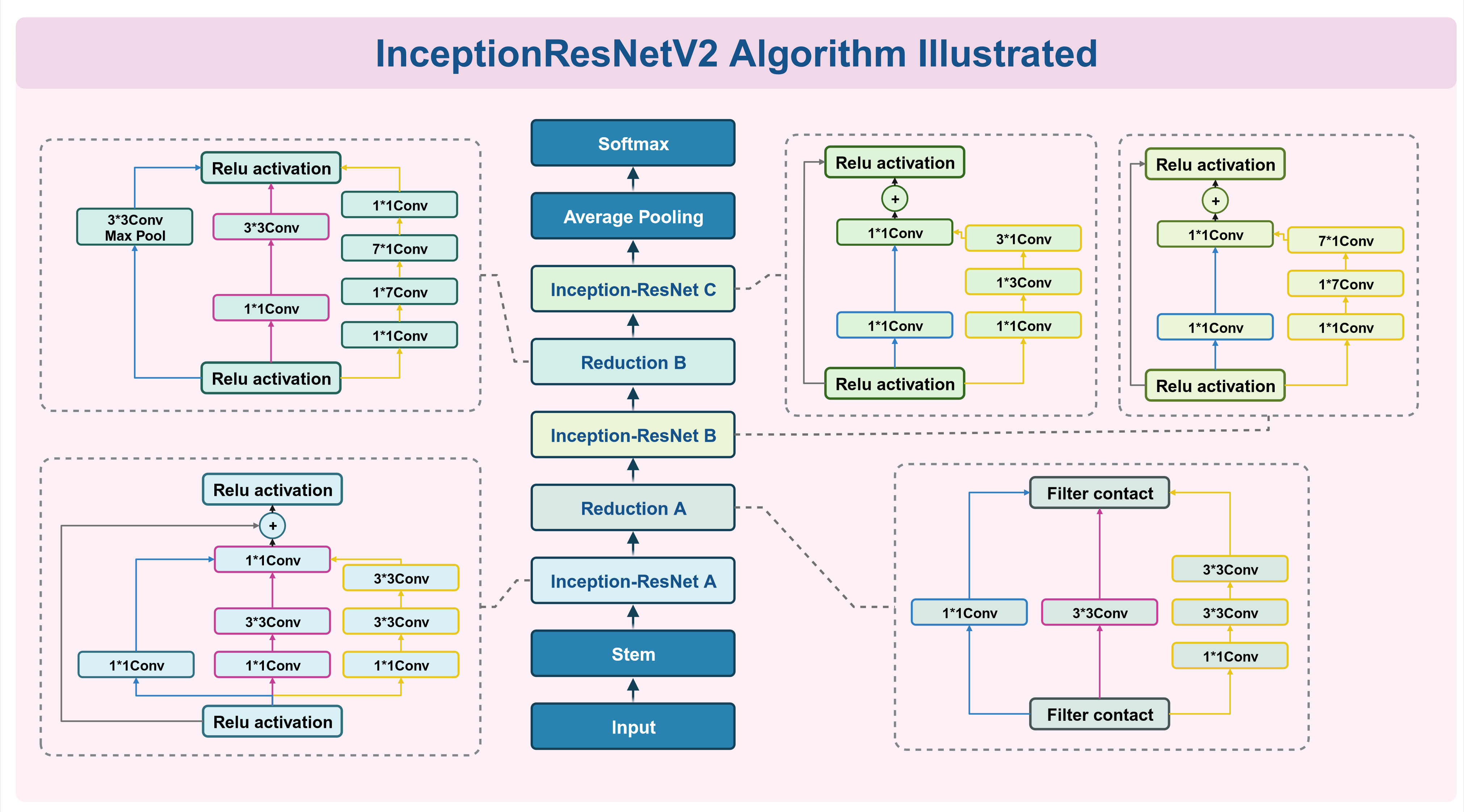

Supplement: Supplementary file 3 [file Image_3.jpeg]

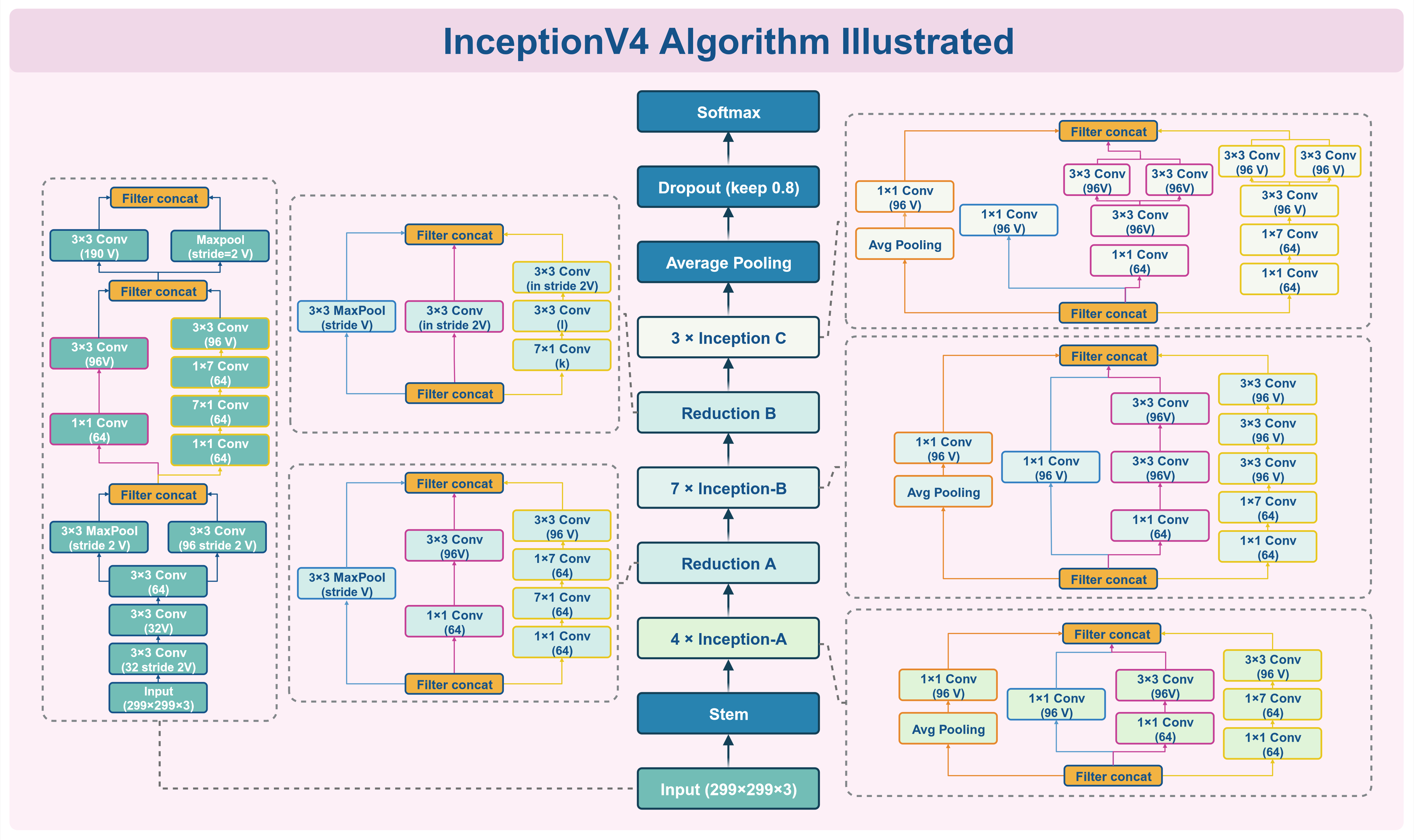

Supplement: Supplementary file 4 [file Image_4.jpeg]

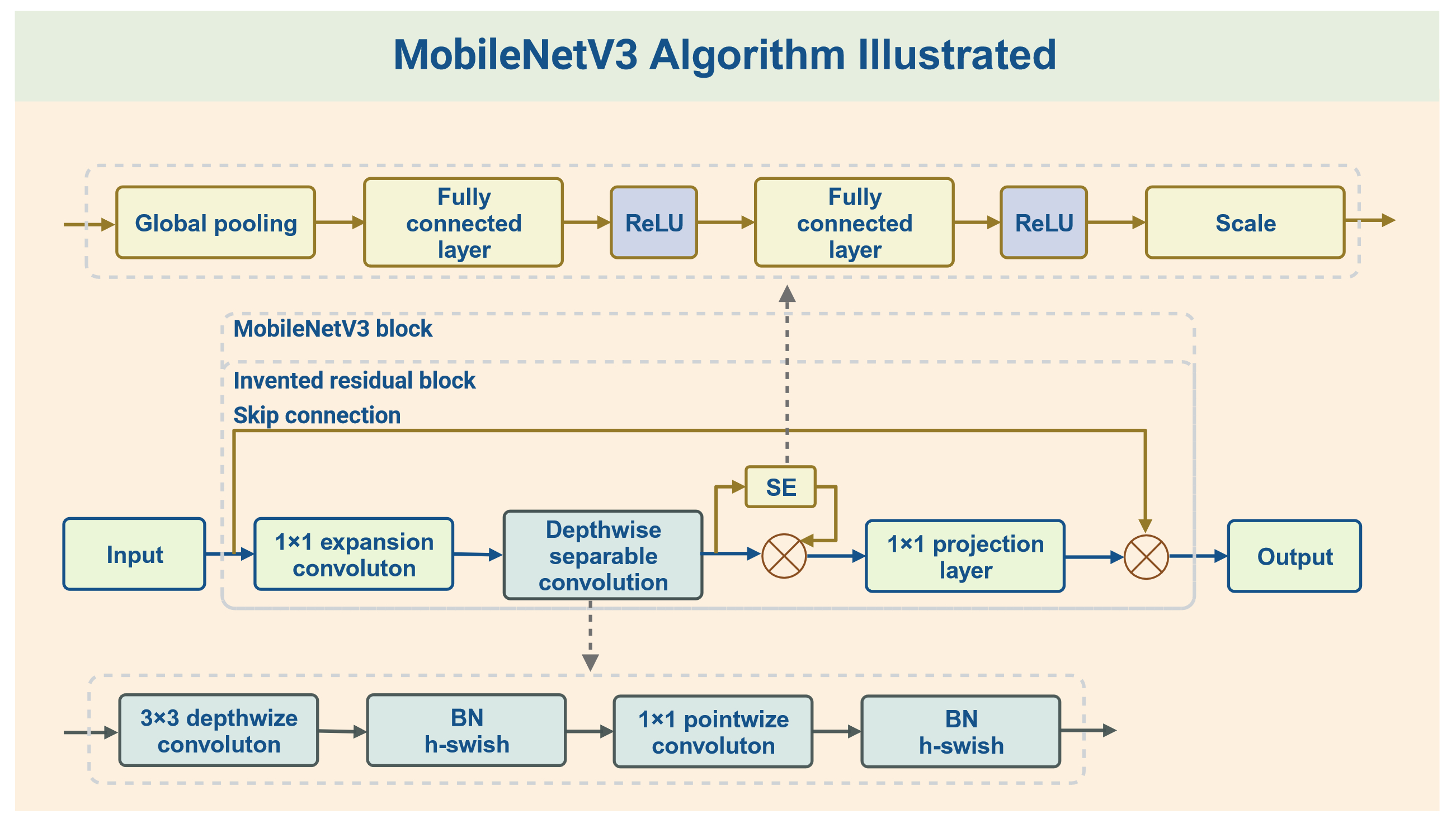

Supplement: Supplementary file 5 [file Image_5.jpeg]

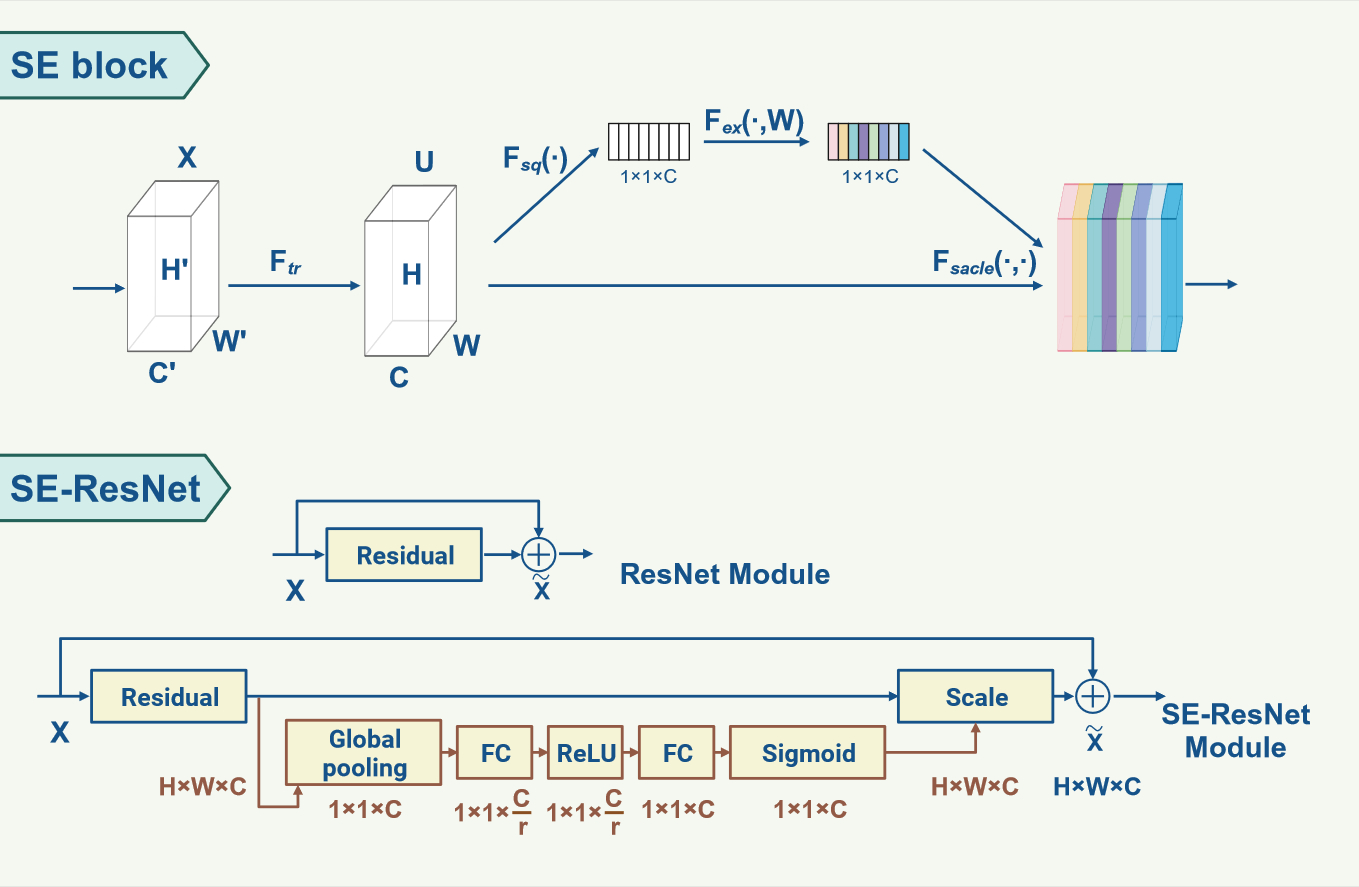

Supplement: Supplementary file 6 [file Image_6.jpeg]

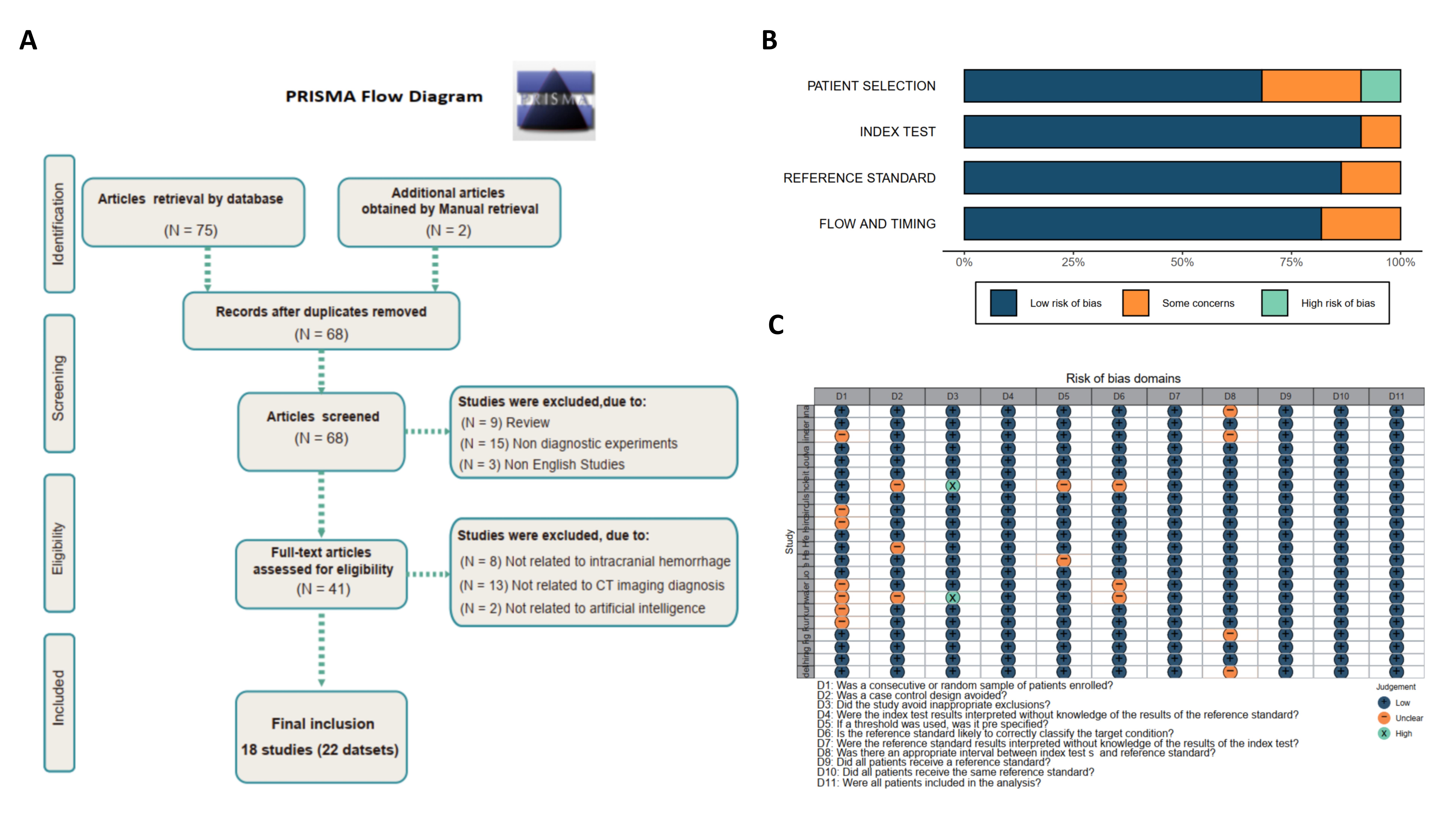

Supplement: Supplementary file 7 [file Image_7.jpeg]

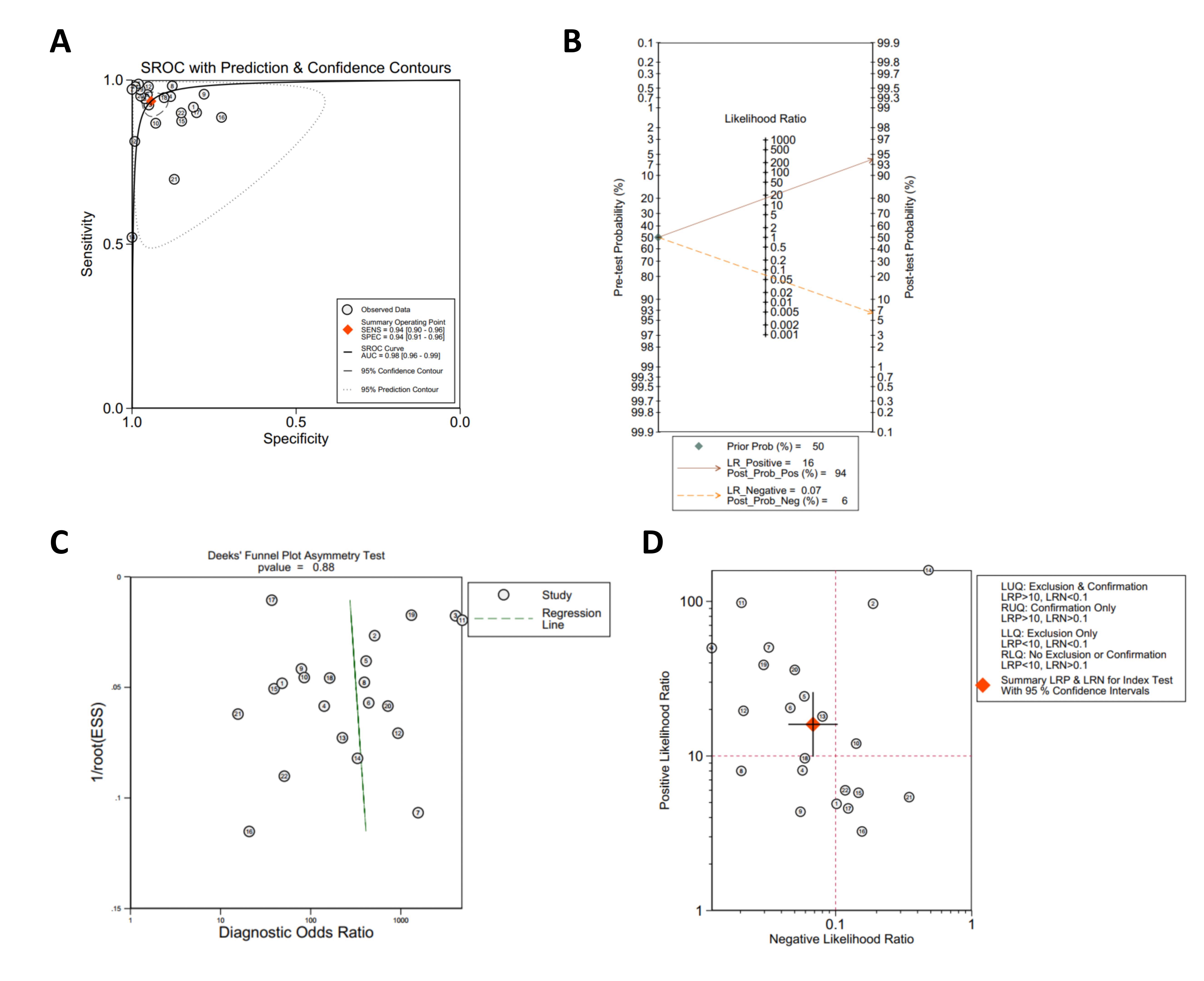

Supplement: Supplementary file 8 [file Image_8.jpeg]

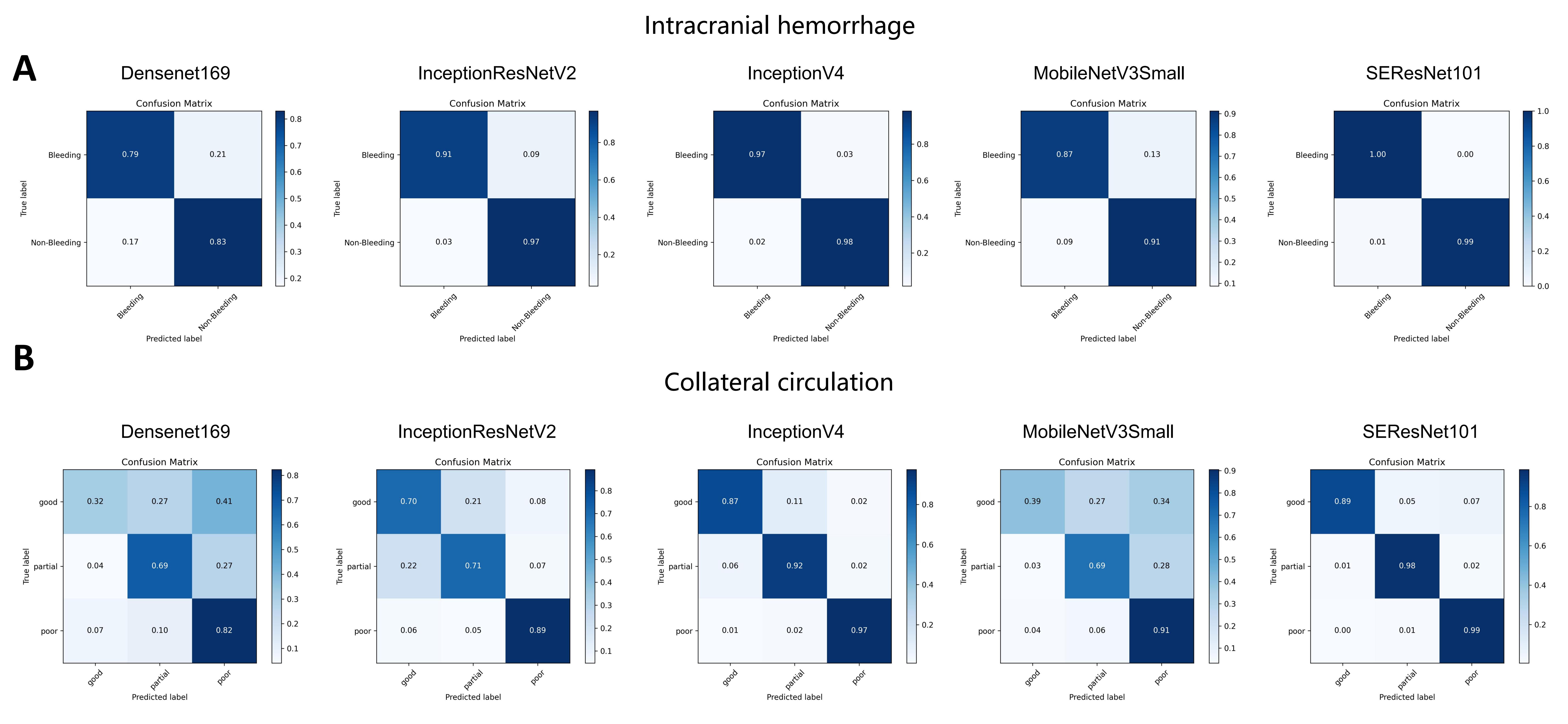

Supplement: Supplementary file 9 [file Image_9.jpeg]
