# Supplementary material for: InceptionV4 and SEResNet101: precise predictors of intracranial hemorrhage and collateral circulation post—ischemic stroke intervention
Source: Front Neurol. 2025 Sep 17;16:1617626. doi: 10.3389/fneur.2025.1617626 (PMC12516262; doi:10.3389/fneur.2025.1617626)
Supplement: Supplementary file 10 [file Table_1.docx]

**Table S1. Sequences of RT-qPCR Primers.**

| **Gene** | **Primer Sequence (5'-3')** |
| --- | --- |
| Kdr mouse | Forward: 5'-TTGCCTGGTCAAACAGCTCA-3'  Reverse: 5'-GCTCTGCTTCCAGGAGTGTG-3' |
| Lcn2 mouse | Forward: 5'-TGTCATGTGTCTGGGCCTTG-3'  Reverse: 5'-AACTGATCGCTCCGGAAGTC-3'' |
| Pxn mouse | Forword: 5'-GACGACCTCGATGCCCTG-3'  Reverse: 5'-CAAGAACACTGGCCGTTTGG-3' |
| Gapdh mouse | Forward: 5'-AAGAGGGATGCTGCCCTTAC-3'  Reverse: 5'-TACGGCCAAATCCGTTCACA-3' |
