# Supplementary material for: InceptionV4 and SEResNet101: precise predictors of intracranial hemorrhage and collateral circulation post—ischemic stroke intervention
Source: Front Neurol. 2025 Sep 17;16:1617626. doi: 10.3389/fneur.2025.1617626 (PMC12516262; doi:10.3389/fneur.2025.1617626)
Supplement: Supplementary file 11 [file Table_2.docx]

**Table S2. Antibody Information for Western Blot.**

| **Target Name** | **Manufacturer** | **Catalog Number** | **Dilution Ratio** |
| --- | --- | --- | --- |
| Kdr (mouse) | Abcam | ab315238 | 1:1000 |
| Lcn2 (mouse) | Abcam | ab216462 | 1:1000 |
| Pxn (mouse) | Abcam | ab32084 | 1:1000 |
| Gapdh (mouse) | Abcam | ab8245 | 1:500 |
