# Supplementary material for: InceptionV4 and SEResNet101: precise predictors of intracranial hemorrhage and collateral circulation post—ischemic stroke intervention
Source: Front Neurol. 2025 Sep 17;16:1617626. doi: 10.3389/fneur.2025.1617626 (PMC12516262; doi:10.3389/fneur.2025.1617626)
Supplement: Supplementary file 12 [file Table_3.docx]

**Table S3. Baseline Feature Table Included in the Literature.**

| **First author** | **CNN model type** | **Country** | **Study design** | **Blinded design** | **Consecutive or random** | **Reference standard** |
| --- | --- | --- | --- | --- | --- | --- |
| Watanabe Y (2021) | CAD system (U-Net) | Japan | R | unknown | Random | 15 physicians (5 board-certificated radiologists, 5 radiology residents, and 5medical interns) |
| Voter AF (2021) | AIDOC system | America | R | unknown | Consecutive | a neuroradiologist |
| Salehinejad H (2021) | CNN (ResNeXt) | Canada | R | unknown | unknown | a neuroradiologist |
| Rava RA (2021) | Canon's AUTO system | America | R | unknown | Consecutive | 2 neuroradiologists |
| McLouth J (2021) | CINA® v1.0 device | America | R | unknown | Random | 2 neuroradiologists |
| Heit JJ (2021) | CNN | America | R | YES | Random | 3 neuroradiology experts |
| Finck T (2021) | CNN | Germany | R | unknown | Consecutive | unknown |
| Buls N (2021) | CNN | Belgium | R | YES | Consecutive | Radiologists |
| Monteiro M (2020)-1 | CNN | Europe | R | unknown | unknown | Clinical radiology reports |
| Monteiro M (2020)-2 | CNN | Europe | R | unknown | unknown | Clinical radiology reports |
| Ye H (2019) | CNN | China | R | unknown | Random | 3 radiologists |
| Lee H (2019)-1 | CNN (VGG167, ResNet-508, Inception-v39 and Inception-ResNet-v2) | America | R | YES | Consecutive | 5 neuroradiologists |
| Lee H (2019)-2 | CNN | America | P | YES | Random | 5 neuroradiologists |
| Kuo W(2019) | CNN | America | P | unknown | Random | 4 radiologists |
| Ker J (2019) | CNN | Singapore | R | unknown | unknown | unknown |
| Grewal M (2018) | CNN | America | R | unknown | unknown | unknown |
| Chilamkurthy S (2018)-1 | CNN | India | R | unknown | unknown | Clinical radiology reports |
| Chilamkurthy S (2018)-2 | CNN | India | R | unknown | unknown | Clinical radiology reports |
| Chang PD (2018)-1 | CNN | America | R | unknown | Consecutive | a radiologist |
| Chang PD (2018)-2 | CNN | America | P | unknown | Consecutive | a radiologist |
| Arbabshirani MR (2018) | CNN | America | P | YES | Random | a neuroradiologist |
| Prevedello LM (2017) | CNN | America | R | unknown | Random | a neuroradiologist |

Note: CNN represents Convolutional neural networks, R represents Retrospective, P represents Prospective.
